# Utterance evolution: the road to generative, combinatorial communicators

**DOI:** 10.1002/brv.70185

**Published:** 2026-05-17

**Authors:** Catherine Crockford, Roman Martin Wittig, Cedric Girard‐Buttoz

**Affiliations:** ^1^ The Ape Social Mind Lab, Institute for Cognitive Sciences, CNRS UMR 5229 University of Lyon 1 43 boulevard du 11 Novembre 1918 Villeurbanne 69622 France; ^2^ Department of Human Behavior, Ecology and Culture Max Planck Institute for Evolutionary Anthropology Deutsche Platz 6 Leipzig 04103 Germany; ^3^ Taï Chimpanzee Project, CNRS IRL2041 ChiMP4IC Centre Suisse de Recherches Scientifiques 01 BP 1303, Adiopodoumé, km 17, route de Dabou, 01 Abidjan Côte d'Ivoire; ^4^ ENES Bioacoustics Research Laboratory, Centre de Recherche en Neurosciences de Lyon, CNRS, Inserm 69500 University of Saint‐Etienne 23 rue du Dr. Paul Michelon Saint‐Etienne 42100 France

**Keywords:** Vocal sequences, call combination, compositionality, syntax, meaning, language evolution, vocal learning, animal communication, bioacoustics, mammals, birds, chimpanzees

## Abstract

Language has long been considered uniquely complex in the animal kingdom; however, animal research over the last decade has begun to challenge some long‐standing premises about exactly which language capacities are uniquely human. The task of resolving why and how complex communication systems evolve, particularly human language, has manifested in a rich multi‐disciplinary field, which relies on accurate empirical and comparative assessment of natural animal communicative capacities. For example, comparative models of relevant gene and brain functions will remain limited without cracking the animal communication code. To achieve this, empirical research needs an updated, quantitative and comparative framework that distinguishes combinatorial systems from simpler ones. We offer such a framework by integrating new work, demonstrating diverse meaning‐bearing combinatorial signalling strategies in natural animal communication, into cross‐disciplinary theoretical frameworks. We detail four major mechanistic transitions which we argue offer both evolutionary and ontogenetic routes from non‐combinatorial communicators, that mainly rely on single signal utterances, to generative combinatorial signal users, that mainly rely on combinatorial, meaning‐based utterances. These transitions are measurable and predictable: (*i*) signal combination versatility; (*ii*) signal combination learning and conventionalization; (*iii*) message expansion through combinatorial mechanism diversity and versatility; and (*iv*) message expansion through syntax involving linear or hierarchical rules. We offer testable hypotheses for each transition and propose that species demonstrating combinatorial Transitions (*i*) and (*ii*) will be prime candidates for testing Transitions (*iii*) and (*iv*), where generativity can emerge. We also point to future methodological tools to assess the meaning of signals and their combinations in signal utterance‐to‐context mapping analyses. Critically, we argue that simply having research on different species is not enough – testing evolution of language theories requires comparative research across species and ontogeny, based on relatable, quantitative measures. To date, only humans demonstrate meaning‐based, generative communication. However, recent discoveries, particularly of combinatorial versatility in chimpanzee (*Pan troglodytes*) and bonobo (*Pan paniscus*) vocal sequences, open the possibility of generative communication in other species. Whether such traits are human‐unique can no longer be assumed but must be tested.

## I. INTRODUCTION

**Fig. 1:**
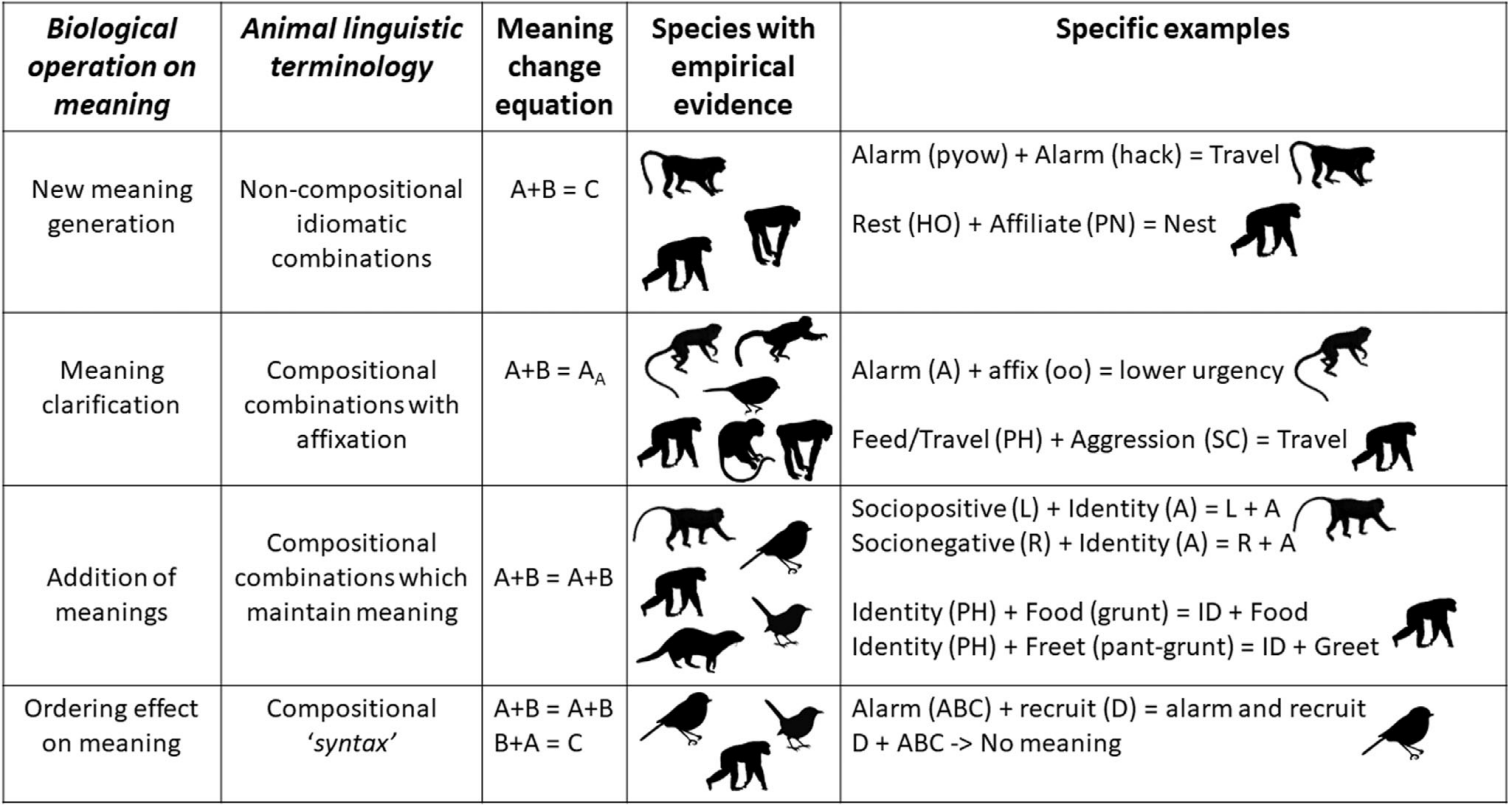
Combinatorial mechanisms documented in non‐human animal natural signalling systems. Each mechanism has so far been reported only in call combinations comprising 2 units (bigrams). The first column states how meaning is modified in bigrams compared to the constituent single units. The second column indicates the linguistic terminology used in the animal communication literature. The third column presents a theoretical formulation of each mechanism. The fourth column uses silhouettes to depict the non‐human animal species for which each phenomenon has been described (from top to bottom and left to right: Putty‐nosed monkeys (*Cercopithecus nictitans*), Arnold & Zuberbühler, 2008; bonobos (*Pan Paniscus*), Berthet *et al*., 2025; chimpanzees (*Pan troglodytes*), Girard‐Buttoz *et al*., 2025; Campbell's monkey (*Cercopithecus campbelli*), Arnold & Zuberbühler, 2006; titi monkeys (*Callicebus nigrifrons*), Berthet *et al*., 2019; black‐capped chickadee (*Poecile atricapillus*), Templeton, Greene & Davis, 2005; Freeberg, 2008; chimpanzees, Girard‐Buttoz *et al*., 2025; olive colobus (*Procolobus verus*), Gallot *et al*., 2025; bonobos, Schamberg *et al*., 2016; Diana monkeys (*Cercopithecus diana*), Coye, Zuberbühler & Lemasson, 2016; Japanese tits (*Parus minor*), Suzuki, Wheatcroft & Griesser, 2016; chimpanzees, Leroux *et al*., 2021; Girard‐Buttoz *et al*., 2022a, 2025; pied babblers (*Turcoides bicolor*), Engesser *et al*., 2016; banded mongooses (*Mungos mungo*), Jansen, Cant & Manser, 2012; Japanese tits, Suzuki *et al*., 2016; pied babblers, Engesser *et al*., 2016 and chimpanzees, Girard‐Buttoz *et al*., 2025). The last column provides specific examples of each mechanism (from top to bottom: Putty‐nosed monkeys (Arnold & Zuberbühler 2008); chimpanzees (Girard‐Buttoz *et al*., 2025), Campbell's monkeys, Arnold & Zuberbühler, 2006; chimpanzees, Girard‐Buttoz *et al*., 2025; Diana monkeys, Coye, Zuberbühler & Lemasson, 2016; chimpanzees, Leroux *et al*., 2021; Girard‐Buttoz *et al*., 2025, and Japanese tits, Suzuki *et al*., 2016. Whilst studies using alarm contexts form the foundation of our understanding of combinatorial systems, recent studies also demonstrate rich combinatorial usage in other contexts, such as during social interactions.

Language has long been considered uniquely complex in the animal kingdom, largely due to its combinatorial and generative nature (Hauser, Chomsky & Fitch, 2002; Fitch, Huber & Bugnyar, 2010; Friederici, 2011; Suzuki, Wheatcroft & Griesser, 2018; Zuberbühler, 2019; Schlenker *et al*., 2023), where words can be endlessly combined and recombined to create an infinite range of new meanings. To claim that traits are uniquely human requires empirical assessment of other animal signalling systems. However, there is a dearth of empirical tests that assess whether other animals possess generative, combinatorial capacities, and with good reason. Decoding animal signal systems is a cumulative process. For example, we need to understand what single signals mean before we can understand the information conveyed by signal combinations. This problem is well illustrated by Hauser *et al*. (2002) who laid out numerous relevant comparative approaches to test which components of the faculty of language animals possess, but none of them explicitly examined combinatorics or generativity in natural animal systems. Recent advances in ethological research in natural animal signal systems allow us to dig into how, how often and how much animals combine signals that modify, add or change meaning (Suzuki, Wheatcroft & Griesser, 2016; Engesser, Ridley & Townsend, 2016; Townsend *et al*., 2018; Suzuki *et al*., 2018; Engesser & Townsend, 2019; Zuberbühler, 2019, p. 218; Girard‐Buttoz *et al*., 2022b; Schlenker *et al*., 2023; Berthet, Surbeck & Townsend, 2025; Girard‐Buttoz *et al*., 2025), revealing unexpected and widespread combinatorial capacities in signalling (Fig. 1). In spite of these advances, no non‐human animal system is considered inherently combinatorial or generative. This is partly due to a lack of integration of empirical and theoretical work, and partly due to difficulties comparing capacities across species. Here, we integrate both recent empirical (reviewed in Schlenker *et al*., 2016, 2024; Townsend *et al*., 2018; Berthet *et al*., 2022) and past theoretical work into a framework which facilitates quantitative comparison across phylogeny and ontogeny. The aim is to aid identification of combinatorial and generative animal signalling systems (Freeberg, Dunbar & Ord, 2012; Manser *et al*., 2014; Townsend *et al*., 2018; Peckre, Kappeler & Fichtel, 2019; Zuberbühler, 2019; Schlenker *et al*., 2023). Throughout this review, we focus on animal signal *combinations*, including vocal or non‐vocal modalities.

**Fig. 2:**
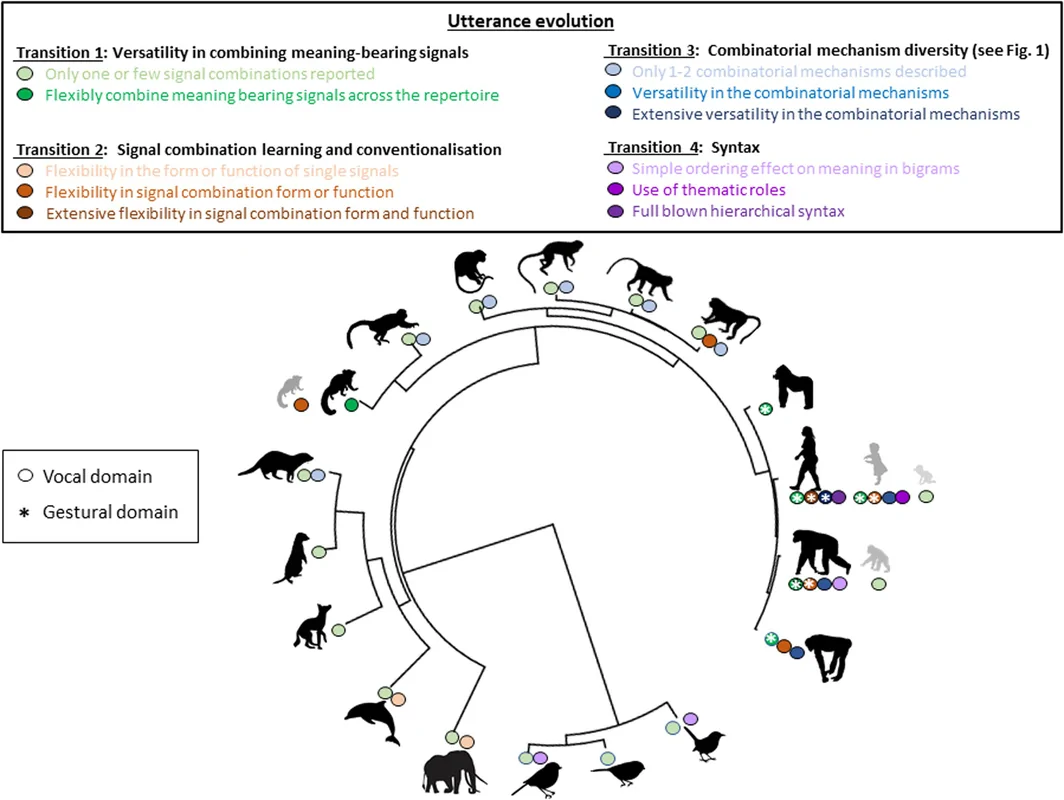
A comparative view of combinatorial signalling capacities across phylogeny and ontogeny in natural animal systems. We define signal combinations as those that demonstrate a shift or addition of meanings compared to the constituent signals. Combinatorial mechanisms describe how meaning changes, such as through meaning addition or modification (see Table 1). For decades, non‐human primates' apparently limited vocal skills have proved a conundrum for theories of how language evolved. Recent evidence puts primates, particularly extant hominids, ‘back in the game’ as they currently show the strongest evidence for language precursors, including generalized capacities to encode meaning within signal combinations, signal usage learning within signal combinations, and vocal sequence expansion through ontogeny. Circle: evidence of skill in vocal modality; asterisk: evidence in gestural modality. Light to dark circles: implicated or demonstrated evidence for limited (light), intermediate (bright) or expanded (dark) capacities. From the bottom and clockwise the species depicted are as follow: Pied babbler (*Turcoides bicolor*), black‐capped chickadee (*Poecile atricapillus*), Japanese tit (*Parus minor*), African elephant (*Loxodonta africana*), bottlenose dolphin (*Tursiops truncatus*), dingo (*Canis lupus dingo*), meerkat (*Suricatta suricatta*), banded mongoose (*Mungos mungo*), marmoset (*Callithrix jacchus*), titi monkeys (*Callicebus nigrifrons*), olive colobus (*Procolobus verus*), Campbell's monkey (*Cercopithecus campbelli*), Diana monkey (*Cercopithecus diana*), Putty‐nosed monkey (*Cercopithecus nictitans*), human (*Homo sapiens*), chimpanzee (*Pan troglodytes*) and, bonobo (*Pan paniscus*). References for the specific studies demonstrating these capacities in each species can be found in the main text and in Fig. 1.

A first step to understanding signal combinations is identifying single signals and their usage. A single signal constitutes a functional communicative unit. This can be a single call, a repetition of the same call type or a sequence of different calls (see Salis, Martin & Girard‐Buttoz, 2025, for a recent discussion on this issue). We define a signal combination in the broad sense, as the combination of at least two different *meaning‐bearing* signals into a longer meaningful structure without presupposition as to the relationship between the meaning of the composing signals and that of the combination (Table 1). Note that this definition differs from some stricter definitions found in the literature where combinatoriality is only characterized if the meaning of the combination is not a simple addition of the meanings of the composing signals (e.g. Scott‐Phillips & Blythe, 2013). Our semantic choice aligns with recent discussion of animal combinatorial communication (Engesser & Townsend, 2019) and allows a more inclusive consideration of animal communicative systems across a variety of taxa (Fig. 2).

**Table 1:** Glossary of terms, operationally defined to enable comparative quantitative analyses.

Non‐combinatorial communicator	Mainly relies on single‐signal utterances.
Combinatorial communicator	Mainly relies on combined utterances that expand meanings such that signal combinations are commonly used across daily life.
Signal sequence	A generic term for signal output containing more than one signal type.
Signal combination	A signal sequence in which at least two different meaning‐bearing units are strung in a sequence to form a meaningful composite.
Combinatorial mechanism	The function by which meaning is changed in a signal combination compared to its constituent parts, such as modifying or adding meanings, or generating new meaning (Fig. 1). Combinatorial mechanisms can give rise to specific rules, such as in English, ‘un‐’ operates as a negation when prefixed to a word like ‘sympathetic’, or in Campbell's monkeys ‘oo’ down‐grades urgency when suffixed to alarm calls. Such mechanisms and rules may or may not overlap with linguistic rules.
Generative	Combinatorial mechanisms or rules that generalize across call types and contexts, such that each can liberally generate new messages or relationships.
Vocal production learning	Adding a new call to one's repertoire through individual or social learning.
Vocal usage learning	Learning to use an existing call in the same context as others use it, in a new context, or in the same sequential order as others use it, through individual or social learning.
Signal form	The shape (or acoustic structure) of a signal.
Signal function	The communicative purpose of the signal, often ascertained through natural contextual usage or experiments.
Conventionalization	Social learning standardizes signal combination meanings within social groups or populations.
Syntax	Linear or hierarchically structured rules or mechanisms governing meaning‐bearing signal combinations.
Non‐adjacent dependencies	Dependencies between nonadjacent elements within a sequence.

What makes language complex is layered flexibility in combining sounds, termed duality of patterning, where meaningless sounds used in speech (phonemes) are combined into words that bear meaning, which are themselves combined into hierarchically organized meaningful utterances (Hockett, 1959). Utterances flexibly describe relations between an endless array of real‐world objects and events, including complex social scenarios (e.g. Stewart, McElwee & Ming, 2013). Most languages use a small set of less than 50 phonemes (Moran, McCloy & Wright, 2012), which overlaps with the number of sounds used in other primate and animal vocal repertoires (see Section II). Hence, we have two expectations for any non‐human animal generative signalling system. First, it will rely on combinatorial capacities rather than large sound sets. Second, learning of signal‐to‐meaning mappings may be evident, and so benefit from ontogenetic assessment.

The vast majority of animal utterances are composed of single calls, such as tweet, chirp, hoo, grunt, or repetitions of the same single call (e.g. Bouchet, Blois‐Heulin & Lemasson, 2013), some of which convey meaning, beyond the signaller's emotional state, to conspecifics about objects or events external to the signaller (see Section II). When such meaning‐bearing calls are combined into call combinations, animal examples to date (Fig. 1) demonstrate that meanings can be (*i*) *modified* such that one call modifies the meaning of the other(s) (Coye, Zuberbühler & Lemasson, 2016; Girard‐Buttoz *et al*., 2025), (*ii*) *added* such that the meaning of the composing calls is preserved (Suzuki *et al*., 2016; Engesser *et al*., 2016; Dutour, Lengagne & Léna, 2019; Girard‐Buttoz *et al*., 2025), or (*iii*) *changed* such that the combination generates an entirely new meaning unrelated to the meaning of the composing calls (Arnold & Zuberbühler, 2008; Girard‐Buttoz *et al*., 2025). We define a combinatorial mechanism as the function by which the meaning of a single signal is altered when strung into a signal combination, that is (*i*), (*ii*) and (*iii*) above are three distinct combinatorial mechanisms (Table 1). Such combinatorial signalling is evident both in species that can learn vocalizations (song birds: tits, Suzuki *et al*., 2016; Dutour *et al*., 2019; magpies, Walsh *et al*., 2019; elephants, Hedwig & Kohlberg, 2024) and in species considered to have more fixed, inherited vocal repertoires [primates: monkeys, Arnold & Zuberbühler, 2006, 2008; Coye *et al*., 2015, 2016; Berthet *et al*., 2019; Gallot *et al*., 2025; great apes, Schamberg *et al*., 2016; Schamberg, Surbeck & Townsend, 2024; Leroux *et al*., 2023; Berthet *et al*., 2025; Girard‐Buttoz *et al*., 2025; carnivores: meerkats (*Suricata suricatta*), Rauber, Kranstauber & Manser, 2020; banded mongooses (*Mungos mungo*), Jansen, Cant & Manser, 2012; babblers, Engesser *et al*., 2016; Figs 1 and 2]. Hence, it is now clear that combinatoriality is a general animal phenomenon used to expand the range of information that can be conveyed beyond the number of available single signals.

However, for most species, signal combinations used are few and short, containing two call types (bigrams). With the exception of chimpanzees (*Pan troglodytes*) (Girard‐Buttoz *et al*., 2025), to date, no more than two combinatorial rules or mechanisms (Table 1; Fig. 1) have been documented per species, and these examples are mostly found in alarm contexts. Hence in most animal systems, whilst combinatoriality is used it does not liberally expand messaging. Critically, rigorous demonstrations of the capacity to combine meanings into utterances to describe relations between *two or more concomitant real‐world objects and events* in animal communication are few (Suzuki *et al*., 2016; Engesser *et al*., 2016; Girard‐Buttoz *et al*., 2022a; Bortolato *et al*., 2023a; Leroux *et al*., 2023; Girard‐Buttoz *et al*., 2025). Until utterances combine meanings, signallers remain highly limited in their capacity to signal about relations between agents, patients and events. Per species, the extent to which combinatorial capacities are *flexibly utilized across daily life* determines generative capacity – and the complexity – of the communication system. Species that are *non‐combinatorial communicators* have no or only few, short signal combinations utilizing only one combinatorial mechanism, and therefore have no generative capacity. In contrast, species which are *combinatorial communicators* would have many, signal combinations of various lengths used liberally across daily life events, and which can utilize several combinatorial mechanisms across call types and contexts (Table 1). Such a system would also have generative potential. Recent research suggests that, to some extent, the combinatorial limitations described per species reflect the limits of research effort rather than limitations in animal signalling combinatoriality (Berthet *et al*., 2025; Girard‐Buttoz *et al*., 2025).

Major evolutionary theories tackle what drivers might promote the evolution of complex communication, such as the social complexity hypothesis for communicative complexity. This hypothesis posits that increasingly complex social life selected for signal complexity to provide more diverse and nuanced messages (Jolly, 1966; McComb & Semple, 2005; Freeberg *et al*., 2012; Manser *et al*., 2014; Peckre *et al*., 2019; Planer & Sterelny, 2021; Grampp *et al*., 2023). This is the case whether or not complexity approaches a human language‐like system. Research examining evolutionary drivers of signalling is hampered by a lack of clarity about what broadly defines or constitutes more *versus* less complex communication (Freeberg *et al*., 2012; Manser *et al*., 2014; Peckre *et al*., 2019), and complex systems more generally (Kappeler, 2019).

We argue that a central tenet of signal complexity should be to expand messaging. In this review, however, we focus not on why, but on *how*, communicative complexity arose. The only known natural system to generate expansive messaging is language. Language evolved as a generative combinatorial system constructed from a small sound set used in speech – and less commonly utilizing a larger signal set in the case of sign language. (Hauser *et al*., 2002; Jackendoff & Wittenberg, 2017; Table 1). Whilst theories of language evolution are numerous, there is strong agreement that critical evolutionary transitions towards language involved acquiring capacities in meaning‐based combinatoriality, signal combination learning, and in the production and comprehension of syntactical, hierarchical structures (Lewis, 1969a; Nowak, Plotkin & Jansen, 2000; Hauser *et al*., 2002; Fitch *et al*., 2010; Skyrms, 2010; Petkov & Jarvis, 2012; Scott‐Phillips & Blythe, 2013; Nowicki & Searcy, 2014; Truswell, 2017; Jackendoff & Wittenberg, 2017; Townsend *et al*., 2018; Engesser & Townsend, 2019; Zuberbühler, 2019; Jarvis, 2019; Planer & Sterelny, 2021). How these components interconnected through evolution remains highly debated, mainly due to a dearth of empirical data to draw on. Insights from recent empirical discoveries in animal combinatorics can re‐focus the theory.

Hence, this review has two goals. First, we propose a framework that specifically focuses on four major transitions that expand meaning‐based signal combinatoriality and signal combination learning. We argue these transitions offer both evolutionary and ontogenetic routes from mainly rudimentary, single‐signal communicators to versatile, combinatorial, generative communicators. Second, we identify key capacities on which to focus future research efforts to identify combinatorial and generative communicators. We point to measures that may facilitate empirical comparative and ontogenetic testing of how combinatorial and generative a given communicative system is. We offer a specific focus on the latter, given that, to date, only humans demonstrate meaning‐based signal generativity.

In non‐vocal modalities, such as gesture and facial expression, or multimodal signalling, examples of meaning‐bearing sequences where combinations change or add meanings are currently scarce (Hobaiter & Byrne, 2011; Graham, Furuichi & Byrne, 2020; Amici, Oña & Liebal, 2024). Hence, we focus on the vocal domain, however, the principles discussed apply equally to sequences of any signal modality. To demonstrate this, we use the term ‘signal’ where suitable, but examples mainly detail vocal signals.

Previous research (Nowak & Krakauer, 1999; Nowak *et al*., 2000; Nowak & Komarova, 2001) has proposed mathematical models of language evolution that assessed the conditions under which combinatorial signalling systems are selected over the use of single signals. These models demonstrated that a generalized combinatorial signalling system is likely to emerge when (*i*) the number of messages to be conveyed considerably surpasses the number of single signals in the repertoire; and (*ii*) the same signal can be reused in numerous combinations. Whilst (*i*) details the selection pressure, the need to communicate about a wider range of meanings in a larger range of daily life events, (*ii*) details a mechanism, the way to generate more signal possibilities within a constrained sound system. Whilst Nowak focused on (*i*), we focus on (*ii*).

Nowak *et al*. expands on (*i*), that an additional selection pressure to increase combinations occurs when fitness benefits are gained from including more than one piece of information per utterance. This may be particularly so when combinations of meanings in an utterance carry more information than the production of each signal independently, such as detailing relational information between agents and events or agents and patients (e.g. Jackendoff & Wittenberg, 2017; Planer & Sterelny, 2021). Here, simply expanding a repertoire of single signals will not suffice. Franke (2016) demonstrates, by using game theory models, that compositional‐like structures can emerge generatively without requiring sophisticated cognition through spill‐over reinforcement learning, although this is disputed by Scott‐Philipps & Blythe (2013). Importantly, a simple combinatorial system, based on linear grammar that maps directly linear order onto semantic roles, as found in certain languages, does not necessitate complex cognitive abilities but can still denotate roles in an utterance such as agent, action and patient, allowing the communication a large array of messages (Jackendoff & Wittenberg, 2017; Truswell, 2017).

**Fig. 3:**
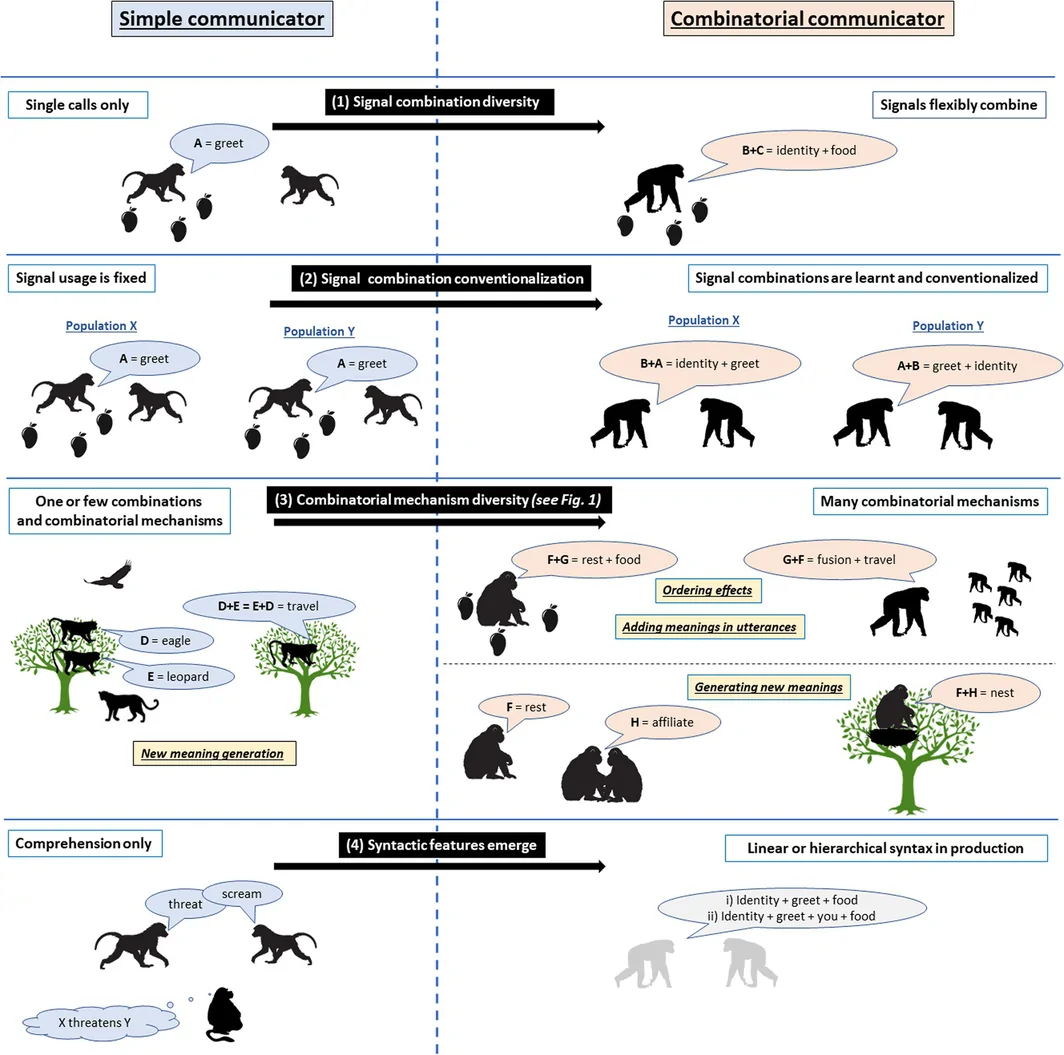
Empirical and theoretical animal examples illustrating four major transitions required to move from simple, non‐combinatorial to combinatorial communicators, across phylogeny or ontogeny. Given the same social scenario, whilst the left column illustrates signalling of a simple communicator, the right column illustrates animals who have reached each respective transition. Signals demonstrate increasing capacity to combine meanings about daily life events within utterances with each transition. We assume combinatorial communicators gain fitness benefits from using signals that encode meaning about numerous and concomitant daily events, leading to utterances which combine multiple meanings. The transitions are as follows: (*i*) signal combination diversity: moving from a reliance on single to combined meaning‐bearing signals; (*ii*) signal combination learning and conventionalization: social learning standardizes arbitrary vocal combination meanings within social groups or populations; (*iii*) diverse combinatorial mechanisms: usage of several combinatorial mechanisms expands expression of daily life events; and (*iv*) syntax: linear or hierarchical structuring of vocal combinations, e.g. referencing thematic roles: (*i*) first person, (*ii*) third person, and combining roles with daily events, e.g. ‘I greet you/him’. Changing the order or adding modifiers may change agent and patient roles. For each transition towards complexity, on the right, we represent empirical (pink speech bubbles) or theoretical (grey speech bubbles) examples of utterances individuals do/might make. Text boxes with yellow backgrounds indicate different combinatorial mechanisms. We use empirical examples for simple, non‐combinatorial communicators: baboons (*Papio ursinus*) (Transitions 1, 2 and 4) (Bergman *et al*., 2003; Maciej *et al*., 2013) and putty‐nosed monkeys (*Cercopithecus nictitans*) (Transition 3) (Arnold & Zuberbühler, 2008); and for complex communicators from Transitions 1–3: chimpanzees (*Pan troglodytes*) (Leroux *et al*., 2021; Girard‐Buttoz *et al*., 2025). For comparability, we draw on empirical examples from the primate literature but several transitions are also evident in other taxa (see Fig. 1 and main text for specific examples). Transition 4, right side, is theoretical, never having been described in a non‐human animal.

Drawing on the social complexity hypothesis, we work with the assumption that as societies become more complex, species gain fitness benefits from communicating about more facets of daily life. Drawing from known or putative examples, these might include expressing concomitant information [e.g. the alarm + recruit vocal combination in Japanese tits (Suzuki *et al*., 2016), rest + feed vocal combinations in chimpanzees (Girard‐Buttoz *et al*., 2025)], or relations between individuals and events [e.g. signaller identity + travel/greet/feed vocal combinations in chimpanzees (Leroux *et al*., 2021; Girard‐Buttoz *et al*., 2022a, 2025)], which are not possible with single‐signal utterances (e.g. Bortolato *et al*., 2023a; Fig. 3). Accrued fitness benefits from utilizing signal combinations may result in further expansion of signal combination usage. Selection then acts to transform signal systems from a dependence on single signals to a dependence on signal combinations. Given the changing nature of how social or ecological events unfold in daily life, genetically fixed signalling systems involving minimal learning are likely to be too constrained to communicate liberally about daily life events. Hence, we expect some evidence that learning shapes the structure and usage of signal combinations.

Given a complex society, which includes most species represented in Fig. 2 (especially large multi‐male–multi‐female groups, cooperative breeding or fission–fusion societies), and drawing on signalling theories (see Section II), we assume (*a*) that there is selection pressure to generate more messages than there are single signal types. In line with Nowak *et al*.'s model, a generative combinatorial signalling system can only emerge if the same signal can be reused in numerous combinations. We expand on this idea by adding that four major mechanistic transitions are required to build such a signalling system (Fig. 3), in which all four transitions are combinatorial, and Transitions 3 and 4 may, but do not have to, include generativity. Whilst transitions 1 to 3 may overlap in their emergence, we expect that species which demonstrate transition 1 and 2 traits are the most likely to also demonstrate transition 3 and 4 traits. Throughout this review, where they exist, we provide empirical evidence of these transitions across the animal kingdom (summarized in Fig. 2).

The first of these transitions is ‘signal combination diversity’ in which single meaning‐bearing signals are flexibly combined. The signal system shifts from a dependence on single signal utterances to reliance on utterances combining meaning‐bearing signals throughout daily life.

In Transition 2, signal combination learning and conventionalization, usage learning of signal combinations and their meanings emerge. Learned arbitrary signal forms and functions must then adopt socially recognized conventions within communities for communication to be successful.

In Transition 3, combinatorial mechanism diversity and versatility, signals that are emitted in short combinations (bigrams) using mechanisms that change or add signal meanings in different ways expand messaging, particularly by combining two meanings within utterances (Table 1; Fig. 1). Each mechanism can be used in several signal combinations using different rules or signals, possibly showing the emergence of a generative system. Versatility results in a system where messaging capacity shows expansion well beyond the available number of single signals such that everyday life events can be related and communicated. Combinatorial capacities are considered generative when they can encode information about numerous daily life events.

The fourth and final transition is ‘syntax’. Combinatorial mechanisms become numerous and are exploited in longer signal combinations (trigrams or longer), containing at least three meanings within utterances. Hence, grammatical rules governing the organization of calls within utterances are required to convey relational information about daily events likely through a combination of linear or hierarchical structuring, which in turn facilitates message generativity.

In sum, studies to date that have examined call combinations have mainly focused on alarm contexts (Fig. 1), such that the range of meanings at the scale of the full signal repertoire is not well documented. Likewise, the versatility with which such meaning‐bearing signals can be combined and recombined within utterances is largely unknown, with the exception of recent great ape and marmoset studies which demonstrate extensive versatility (Bosshard *et al*., 2024; Berthet *et al*., 2025; Girard‐Buttoz *et al*., 2025). Below, we detail the four major mechanistic transitions we propose, which we argue offer both evolutionary and ontogenetic routes from mainly rudimentary, single‐signal communicators to versatile, combinatorial, generative communicators. We detail how these transitions are measurable and predictable. Critically, we argue that simply having research on different species is not enough – research across species and ontogeny will be more powerful when directly comparable, using relatable, quantitative measures.

## II. SIGNAL COMBINATIONS

The emphasis in this paper is on signal combination production as signal comprehension has been extensively covered elsewhere (e.g. Fitch & Friederici, 2012; Wilson *et al*., 2020). However, as signals produced can only be functionally relevant if they are comprehended, this literature is briefly summarized in below.

### (1) Signal meaning

For a communication system to evolve, signals must provide information that elicits receiver responses that benefit both signaller and receiver (Laidre & Johnstone, 2013; West *et al*., 2015). For instance, aggressive signals must generally work to repel, and copulation signals to attract, and coordination signals to coordinate. This is the case whether or not signals elicit mental representations or reflect the signallers' intentional goals, unintentional actions or simply the signaller's emotional state. In animal systems, determining the exact message encoded in an utterance is nonetheless challenging (Cheney & Seyfarth, 1990; Rendall, Owren & Ryan, 2009; Seyfarth & Cheney, 2017; Fischer & Price, 2017; Watson *et al*., 2022; Amphaeris *et al*., 2023) and has led to extensive interdisciplinary discussion about whether the information conveyed by animal signals to conspecifics constitutes meaning. Typically, repeated behavioural observations of natural signal production in specific contexts that lead to specific behavioural outcomes allow signal‐to‐event mapping needed to delineate species‐specific call types and their potential meaning or function (Seyfarth, Cheney & Marler 1980; Elie *et al*., 2024).

The delineation of what constitutes a single call type and its relevance as an informative communicative signal in itself was then tested *via* carefully designed playback experiments that elicit relevant behavioural responses in the *receiver* in the absence of the associated context in which the call is usually elicited. Foundational studies ingeniously focused on alarm contexts to provide an external referent, the predator, facilitating discussions relating to mental representation and meaning (Seyfarth, Cheney & Marler, 1980; Fischer, 1998; Zuberbühler, Cheney & Seyfarth, 1999). Evidence of highly specific signal‐to‐context mapping across a range of species and contexts has led recent studies to conclude that meaning is an appropriate term when referring to the information contained in animal signals (Sievers & Gruber, 2016; Seyfarth & Cheney, 2017; Zuberbühler, 2019; Berthet *et al*., 2022; Watson *et al*., 2022; Amphaeris *et al*., 2023; Elie *et al*., 2024; Salis *et al*., 2025).

We argue that examining shifts in contextual usage in call combinations compared to constituent calls offers an additional avenue to parse whether signallers are expressing contextual information or merely their emotional state. For example, chimpanzees emit screams when being attacked, a highly emotional context with negative valence. The other dominant context in which chimpanzees emit screams is in combination with a pant hoot vocalization during travel and food contexts (Girard‐Buttoz *et al*., 2025), resulting in contact or reunion with others, particularly allies (Mitani & Nishida, 1993; Crockford, 2019). Hence, the function of a scream may loosely relate to recruitment across contextual usage, but in this example, it cannot be underwritten by the same emotional or arousal state. Rather, contextual information appears dominant to emotional information. Here, the characterization of the call combination and its meaning is possible because of a fine‐grained knowledge of both signal form and function acquired over decades of research. Whether animal calls are analogous to human phonemes like [m] or [o], morphemes like ‘mon‐’ or ‘‐key’, words like ‘monkey’, and/or non‐verbal sounds like laughter (Anikin *et al*., 2023), remains an open question, largely because we do not know enough about how signal combinations operate across whole repertoires. However, the combinatorial scream example, suggests not all call elements are analogous to laughter, as laughter does not fundamentally change its contextual information when combined with other sounds or words.

### (2) Signal intention

Whether the signaller intends to change another's behaviour or mind through the use of a signal has also been a topic garnering intense research. Evidence, particularly in great apes, indicates that using signals with some voluntary control and intent to change behaviour is likely widespread in both vocal and gestural modalities of single signals (Great apes, Hobaiter & Byrne, 2011; Schel *et al*., 2013; Taylor *et al*., 2022; Bouchard & Zuberbühler, 2022; Monkeys, Silk, Seyfarth & Cheney, 2016; Cheney & Seyfarth, 2018). Hence, arguments that signals are only arousal‐driven likely miss crucial complexity. Signal use with apparent intent to change others' knowledge states has been argued for chimpanzees and bonobos (*Pan paniscus*) (Crockford *et al*., 2012; Crockford, Wittig & Zuberbühler, 2017; Schel *et al*., 2013; Genty & Zuberbühler, 2014; Moore, 2016; Girard‐Buttoz *et al*., 2020; Heintz & Scott‐Phillips, 2023; Townrow & Krupenye, 2025), giving insight into how animals think. Whether such signals are ostensive, however, remains untested. In contrast, human speech is considered to be ostensive, such that the signaller not only intends to change another's behaviour or mind but intends for the receiver to be explicitly aware of this intention (e.g. Moore, 2016; Graham *et al*., 2019; Heintz & Scott‐Phillips, 2023). It is assumed that non‐human animals do not engage in ostensive communication, partly because no test of ostension has ever been conducted, although test designs have been proposed (Moore, 2016). However, a new study testing and demonstrating metacognition in chimpanzees suggests meta‐awareness tests in the domain of communication may be feasible (Schleihauf *et al*., 2025).

### (3) Comprehension of natural and artificial signal sequences

Several species tested in captivity with artificial and meaningless sequences demonstrate understanding of both adjacent and non‐adjacent grammatical dependencies (see Petkov & Jarvis, 2012; Fitch & Friederici, 2012; Wilson *et al*., 2020, for reviews), suggesting some capacity to recognize simple non‐adjacent grammatical rules (see Table 1). Non‐adjacent grammatical rules that organize meaning in sentences are critical features of language Fitch & Friederici, 2012. However, this has been demonstrated neither in signal comprehension nor production in the natural communication system of any non‐human animal (Zuberbühler, 2020).

However, relational meaning can be extracted from natural vocal sequences of two adjacent call types beyond the examples in Fig. 1, when the sequences are formed by more than one signaller. Playback experiments demonstrate that baboons keep track of the vocalizations of others (Crockford *et al*., 2007), interpreting them as third‐party interactions. For example, if baboons hear an aggressive threat call from one individual and a scream from another, baboons react more if the threatening individual is subordinate to the screamer (Bergman *et al*., 2003). This suggests that baboons extract a meaningful interaction from the two calls emitted by different signallers, here inferring a relationship between these two calls and callers that indicates that a rare rank change has occurred (Bergman *et al*., 2003; Cheney & Seyfarth, 2018). Given that baboons rarely emit combinatorial utterances (Boë *et al*., 2017; Hammerschmidt & Fischer, 2019) suggests that the proclivity to extract combined meaning from juxtaposed signals from different signallers is more common than the capacity to produce utterances that express combined meaning. In sum, such results have led to the conclusion that comprehension complexity outstrips production complexity, mirroring child language development. Critically, the assertion that hierarchical syntax is a human Rubicon remains (Seyfarth & Cheney, 2010).

## III. TRANSITION 1 – SIGNAL COMBINATION VERSATILITY: SEQUENCES SHIFT FROM CONTAINING SINGLE SIGNALS TO FLEXIBLY COMBINING MEANING‐BEARING SIGNALS

If a species only uses one or two signal combinations when communicating, it suggests that any fitness benefits gained from combining signals do not generate sufficient selection pressure to promote the evolution of a generalized combinatorial communication system, which likely requires commensurate investment in relevant brain infrastructure. A particular challenge may be connecting motor and vocal brain regions with conceptual semantic regions, which, for the human language network, are in different lobes and connected by large white matter pathways (Gallardo *et al*., 2023; Fedorenko, Piantadosi & Gibson, 2024; Becker *et al*., 2025). In the absence of such selection pressures, a versatile production system that combines meanings and can detail an endless array of daily life events, including complex social scenarios, like speech does, seems unlikely to emerge.

Studies initially assessed call combinations in alarm contexts. However, a range of species now also demonstrate meaning‐bearing calls are combined in other contexts (great apes, Leroux *et al*., 2021; Girard‐Buttoz *et al*., 2022a; Bortolato *et al*., 2023a; Berthet *et al*., 2025; monkeys, Bouchet *et al*., 2013; Coye *et al*., 2016; Bosshard *et al*., 2024; elephants, Pardo *et al*., 2019; meerkats, Rauber *et al*., 2020; and mongooses, Jansen *et al*., 2012). These examples across distantly related taxa demonstrate the capacity to convey up to two meanings per utterance. There is, however, as yet no conclusive demonstration that an animal species can convey three or more meanings per utterance, even if this possibility does not appear out of reach for species that recombine bigrams into trigrams and longer sequences like chimpanzees and marmosets (Girard‐Buttoz *et al*., 2022b; Bortolato *et al*., 2023a; Bosshard *et al*., 2024). The latter requires combinations of at least three meaning‐bearing calls or signals, for which there are also few documented examples across species (Bortolato *et al*., 2023a; Bosshard *et al*., 2024; reviewed for primate vocal sequences in Girard‐Buttoz *et al*., 2022b). This is clearly not due to articulatory constraints, given that song, which often requires rapid articulatory transitions (Elemans *et al*., 2008), is widespread across animal taxa, including in some primates. This leans to the possibility of cognitive constraints in semantic encoding, either at the level of comprehension of combined information units (see Section II) or at the production level of encoding meanings sequentially or hierarchically within a single utterance, points also raised by Planer & Sterelny (2021).

A prerequisite of being able to flexibly combine meaning‐bearing calls is having the versatility to flexibly combine call types across the vocal repertoire. Studies that have examined whole repertoires at the species level typically find that 0–30% of utterances are emitted as sequences, and the diversity of sequences is limited given all possible sequences (see Girard‐Buttoz *et al*., 2022b for a review of primates; also see Fig. 5). To date, particularly great apes but also marmosets have been shown to flexibly combine calls into sequences and emit these sequences throughout a wide range of daily life events, not only in rare alarm contexts (Hedwig *et al*., 2014; Leroux *et al*., 2021; Girard‐Buttoz *et al*., 2022a, 2025; Bortolato *et al*., 2023a; Bosshard *et al*., 2024; Schamberg *et al*., 2024; Berthet *et al*., 2025). For example, each third utterance adult chimpanzees emit is a vocal sequence, and most call types combine with most other call types, rendering hundreds of versatile but structured sequences (Girard‐Buttoz *et al*., 2022b). Thus, vocal sequences are common, emitted throughout daily life during numerous events and social interactions. Of the 16 bigrams so far assessed to determine if sequences change the meaning of composite calls, 11 demonstrate clear meaning shifts. Assessing meaning content of repertoires with many sequences is a major task and is on‐going. However, repertoires with a diverse range of sequences across daily life are exactly the repertoires that are the most interesting for assessing the diversity of messaging.

**Fig. 4:**
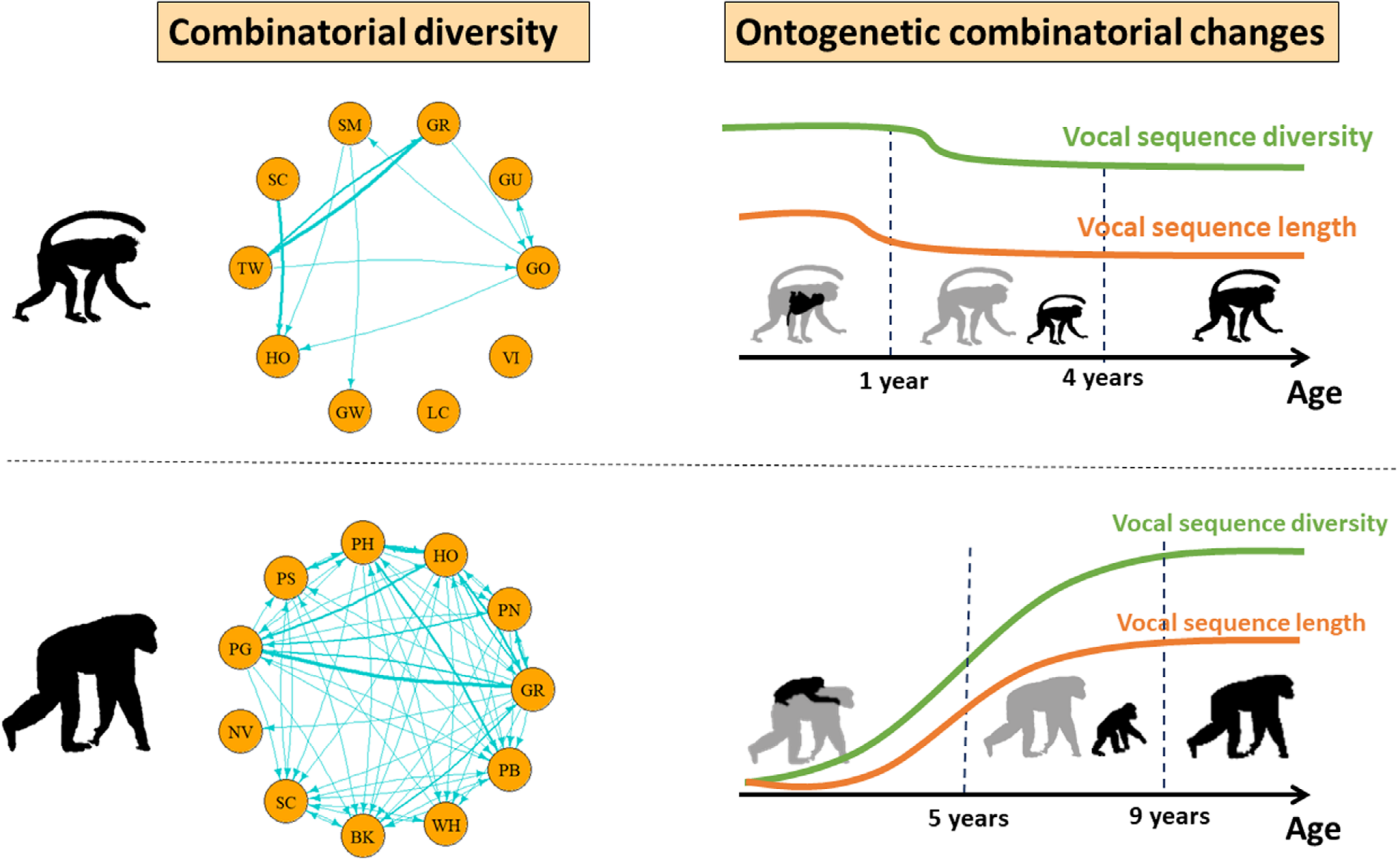
Comparative quantitative measures of combinatorial capacities highlighting variation across species and ontogeny. The networks on the left represent all vocal bigrams (two‐call combinations) documented between single call types at the scale of the full repertoire in adult sooty mangabeys (*Cercocebus atys*; above) and chimpanzees (*Pan troglodytes*; below), data published in (Girard‐Buttoz *et al*., 2022b; Sigmundson *et al*., 2025; Le Floch *et al*., 2026a). These networks illustrate higher call combination diversity in chimpanzees compared to sooty mangabeys. Orange circles depict single call types, and blue arrows show how calls are combined in bigrams. The width of the arrow is proportional to the number of occurrences of each bigram. The right panels, ontogenetic combinational change, illustrate schematically the changes in vocal sequence length (orange) and diversity (green) across ontogeny in those two species [schematic curves redrawn from empirical data published in Bortolato *et al*. (2023b) and Sigmundson *et al*. (2025)]. The individual developmental stage is illustrated with a black silhouette and its mother with a grey silhouette. Whereas both combinatorial metrics remain relatively stable across the lifespan in mangabeys, chimpanzees experience a dramatic increase in both vocal sequence length and diversity across the first decade of life. This comparative example illustrates the value of quantitative whole repertoire approaches for phylogenetic and ontogenetic comparisons.

We hypothesize that an early major transition towards a complex communication system is versatility in combining meaning‐bearing signals, enabling most signals to combine with most other signals throughout a variety of daily life events, and not only in rare events (e.g. alarm). Given that research determining signal meaning is time‐consuming, candidate species for combining meaning‐bearing signals can be detected by assessing species that commonly emit any signal sequences, whether meaning‐bearing or not, throughout numerous daily life events, and that sequence production is not limited to only one or two contexts. Such species would be prime targets to test for the subsequent major transitions required for complex communication detailed below. In contrast, animals that only emit sequences in few contexts can be excluded as candidates. Species can be quantitatively placed along a continuum, aiding both phylogenetic and ontogenetic comparison, by using simple metrics such as utterance length (number of different signals combined within one utterance) and diversity (number of different signal combinations) (see Fig. 4 for empirical examples).

## IV. TRANSITION 2 – SIGNAL COMBINATION LEARNING AND CONVENTIONALIZATION: SOCIAL LEARNING STANDARDIZES NON‐INNATE COMBINATIONS AND THEIR MEANINGS WITHIN COMMUNITIES

Most animal signals are largely genetically fixed (reviewed in Fedurek & Slocombe, 2011; Fischer & Hammerschmidt, 2020) in both their form (the shape of the signal) and their function (the usage of the signal and the outcome it produces) (Table 1). As such, individuals of a given species will emit the same or very similar signal forms to similar stimuli, even in geographically or genetically distant populations, excluding the possibility of social learning (Mitani, Hunley & Murdoch, 1999; Catchpole & Slater, 2003; Crockford *et al*., 2004, p. 2018; Fischer & Price, 2017; Hammerschmidt & Fischer, 2019). Much rarer are signals or their combinations that demonstrate population‐level *meaning shifts*, showing an apparent ‘decoupling of the direct, one‐to‐one linkage between meaning and form’ (Dingemanse *et al*., 2015, p. 610; Watson *et al*., 2022). Such a decoupling suggests an element of learning, where learning can then lead to an arbitrary connection between a signal and its meaning. To become communicatively useful, signal‐to‐meaning mappings need to be shared across the community of communicators (e.g. family, region, country) through social learning, a process referred to as conventionalization (Lewis, 1969b; Moore, 2013). In an arbitrary system, such as human language, the signal form is a symbolic representation of the semantic content it relates to. Learning occurs for both the signal form (‘ape’ takes the form ‘Menschenaffe’ shifting from English to German), and the signal function, contextual usage or semantic content (‘ape’ may change from referring to a particular animal to the action of imitating an ape: ‘to ape’). Given that only humans are thought to have an arbitrary signalling system, the challenge of how species may shift from innate to arbitrary signalling remains empirically unresolved. In linguistics, signal conventionalization is defined as requiring signaller insight into any community‐wide agreement on a particular signal meaning (e.g. Lewis, 1969b). Given early child language‐learning, Moore (2013) argues more simply that signallers are only required to learn that a signal can achieve a particular function, and to imitate the form and usage of the signal. Here, neither insight nor meta‐representation are required to achieve conventionalized signalling through conformity. Conventionalization does not require vocal production learning; it can emerge through social usage learning. Below, we explain why – unlike vocal usage learning – vocal production learning offers limited potential for acquiring new meanings. We then explore how vocal usage learning, especially the learning of signal combinations, can facilitate meaning change and conventionalization. Finally, we emphasize the importance of tracking signal acquisition and usage across development to assess the potential for vocal usage learning.

### (1) Vocal production learning rarely leads to changes in meaning

In the vocal domain, generating entirely or relatively new signals requires vocal production learning (Janik & Slater, 2000; Vernes *et al*., 2021). Vocal learners, like parrots, songbirds, whales and dolphins, are adept at this. In their natural communication system, their strong vocal production learning capacities seem to function to generate group or individual identifiers that mostly serve three functions (within group identification, territorial defense or mate attraction (Janik & Slater, 2000; Catchpole & Slater, 2003; Bradbury & Balsby, 2016); but see Loning, Griffith & Naguib, 2024a). As such, vocal production learning capacities rarely lead to the acquisition of new meanings (Petkov & Jarvis, 2012; Loning, Griffith & Naguib, 2024b). In some animals (elephants, Pardo *et al*., 2024; marmosets, Oren *et al*., 2024; dolphins, King & Janik, 2013) signallers adjust the acoustic properties of their signals to the recipient they address, but the function of the call type itself (an identifier) does not change. In sum, whilst vocal production learning has undoubtedly evolved many times, cases are rare where such learning leads to an expanded capacity to communicate meaning (Berwick *et al*., 2011; Petkov & Jarvis, 2012; Jarvis, 2019). Future research on vocal sequences in these species may yet demonstrate otherwise.

### (2) Usage learning may more readily lead to capacity for meaning change and conventionalization

Changing the signal function or meaning requires vocal usage learning, the capacity to emit an existing sound in a different or new context or to learn to combine signals in sequence (Janik & Slater, 2000; Vernes *et al*., 2021). Identifying the use of arbitrary signalling would require identifying a vocalization that is given a new meaning, which bears little or no intrinsic resemblance to the original meaning (Lewis, 1969b; Moore, 2013; Planer & Kalkman, 2021; Gasparri *et al*., 2023), for example, by observing population or ontogenetic differences in usage. In humans, whilst both vocal production and usage learning are required to become proficient speakers (Vernes *et al*., 2021), given that all human languages construct words from a limited pool of 150 possible phonemes (Moran *et al*., 2012), the question of the extent to which speech actually relies on vocal production learning rather than vocal usage learning is raised. Whilst production learning requires novel sound generation, usage learning requires novel ways to use existing sounds, such as through combinatorial processes.

Usage learning examples to date observed in natural animal systems usually involve a limited contextual shift of single signal use, such as monkey predator alarm calls being elicited by a narrower range of predator types through ontogeny (Seyfarth & Cheney, 1980; León *et al*., 2023) or by novel object presentations with overlapping properties to those of the predator, such as drones eliciting aerial predator alarm calls (Wegdell, Hammerschmidt & Fischer, 2019). Exceptions are the ‘language‐trained’ apes and birds, which produce sequences using human‐made arbitrary signalling systems (bonobos, Savage‐Rumbaugh *et al*., 1993; gorillas, Tanner & Perlman, 2017; African grey parrots (*Psittacus erithacus*), Pepperberg, 2021). In general, once individuals can flexibly combine their calls to create novel sequences, they can either emit novel idiosyncratic sequences, produced only by themselves or copy the sequences of others. Meaning then needs to be mapped onto novel sequences through vocal usage learning processes (Vernes *et al*., 2021).

Examples evoking usage learning involving *signal combinations* in natural animal repertoires are evident in great apes. A bonobo call combination indicates a shift in function, where the bigram whistle‐hoot shows a contextual shift across two wild populations (Schamberg *et al*., 2024). Also, a wild chimpanzee call combination shows population shifts in call order. Here, the bigram pant hoot + pant grunt emitted in fusion + greeting contexts is produced as pant hoot first in a population of western chimpanzees and as pant‐grunt first in an eastern chimpanzee community (Girard‐Buttoz *et al*., 2022a). The contextual usage is consistent across populations but the respective usage of call order has become standard within populations. Finally, in zoo chimpanzees, an oral‐articulated ‘raspberry’ sound can be added *de novo* into a vocal sequence and then adopted by community members (Marshall, Wrangham & Arcadi, 1999), also suggesting propagation through social learning. Of note, in the gestural modality, great apes also show shifts in both form and function, albeit only documented in single signals (Badihi *et al*., 2023a; Malherbe *et al*., 2025; reviewed in Kalan, Nakano & Warshawski, 2025). A number of studies also point to a degree of voluntary control, especially in great ape gestural and vocal signalling (see Section II). Whether this variety of examples indicating usage learning suggests some shift in communicative capacities in hominids or simply under‐reporting in other species requires further research.

### (3) Ontogenetic assessment of signal learning

Across ontogeny, signal combination learning capacity can be assessed by comparing the length and diversity of signal combinations with age (Fig. 4), as well as signal ordering and usage. Learning capacity can be ruled out if infants emit the full repertoire of adults. For single signals, primate cross‐fostering studies between closely related macaque species have demonstrated strong call innateness, as infants only emitted calls of their genetic rather than foster species (Owren *et al*., 1993). To assess whether vocal sequence production is similarly innate, studies have assessed vocal sequence change across ontogeny. Sooty mangabeys (*Cercocebus atys*), a catarrhine monkey, emit over 40 different sequences, but only three are commonly used across individuals (Le Floch *et al*., 2026, 2026). Critically, adults do not have longer sequences, nor a greater diversity of vocal sequences than their infants (Sigmundson *et al*., 2025; Fig. 4), suggesting limited scope for vocal sequence learning. The common marmoset (*Callithrix jacchus*), a platyrrhine monkey that utters a large diversity of vocal sequences across contexts which shift with age, in contrast, shows potential for learning the usage of certain vocal sequences. However, whilst sequence diversity increases in subadults, it shows similarities in infant and adult phases (Gultekin *et al*., 2021). Potential for usage learning of vocal sequences seems higher in chimpanzees, where vocal repertoires expand three‐fold across the first decade of life, shifting from rudimentary utterances of mostly single calls to widespread use of a diverse range of vocal sequences as adults (Bortolato *et al*., 2023b; Fig. 4). Whilst these patterns may be explained by maturational processes, when viewed together with a chimpanzee study showing population variation in vocal sequence structure (Girard‐Buttoz *et al*., 2022a), they suggest social learning may be explanatory for at least some chimpanzee vocal sequences.

In sum, examples in the natural communication systems of great apes suggest that there is sufficient flexibility in signal form and function to warrant testing for the existence of conventionalization processes. This raises two possibilities. First, conventionalization processes arise even with extremely limited usage flexibility such that most of the repertoire remains fixed, or second, there is more usage flexibility in these systems than has been detected to date. Nonetheless, no species other than humans is known to use arbitrary signals with conventionalized signal meanings (Skyrms, 2010; Planer & Sterelny, 2021). It may well be that switching to a conventional system creates demands on pragmatic interpretation, as conventions are likely harder to interpret, generating barriers to their evolution (Aussems & Moore, 2025).

We hypothesize that species that demonstrate both the use of a large diversity of signal combinations (Transition 1) and the decoupling of single signal form and function (Transition 2) are the most likely to demonstrate conventionalization of signal combinations at the level of the social group. We predict that species which use call combinations that particularly demonstrate shifts in function across populations are prime candidates. We do not expect vocal production learning capacities to be influential with respect to a species capacity to be a vocal usage learner. This is because adept compared to more limited vocal production learners do not show greater capacity to embed meaning into utterances, nor in vocal usage learning.

## V. TRANSITION 3 – COMBINATORIAL MECHANISM DIVERSITY AND VERSATILITY: USAGE OF SEVERAL COMBINATORIAL MECHANISMS EXPANDS THE EXPRESSION AND COMBINATION OF DAILY EVENTS

Once a system is in place that can flexibly combine sounds, strong selection pressure to expand the number of messages that can be conveyed is likely required to shift signalling systems to liberally encode combined meanings. It seems unlikely that such a system would emerge to expand messages only about rare events, such as alarm events. More likely, such an emergence would occur when conveying messages that combine information about numerous daily events becomes advantageous (Nowak & Krakauer, 1999).

We argue that a feature particularly likely to promote investment in a combinatorial system is the need to convey concomitant information about everyday events, such that more than one meaning is needed per utterance, such as to encode relational information between actors and/or events. This requires a signalling system (*i*) that liberally combines at least two meaning‐bearing signals within an utterance, (*ii*) where the signal form or function are not necessarily innate but may be learned, (*iii*) leading to short utterances which routinely convey more than one piece of information.

Several bird species [pied babblers (*Turcoides bicolor*) and at least one species of tit (*Parus minor*), Suzuki *et al*., 2016; Engesser *et al*., 2016; Dutour *et al*., 2019] each produce a structured call combination that signals about two synchronous contexts/events – alarm and recruitment (Fig. 1). Whilst impressive, there is only one example per animal repertoire related to a single external stimulus (the presence of a predator). However, there are many occasions throughout an animal's daily life when synchronous events occur, such as greeting an individual in a food patch (Fig. 3). Language can effortlessly incorporate such juxtapositions of events into an utterance e.g. “Hi, let's eat”. But can other animals? We argue that when faced with two synchronous events, a combinatorial communicator will communicate about both in one utterance, whereas a simple, non‐combinatorial communicator will only communicate one event, such as greeting rather than food. However, for most species, at most only a few vocal sequences have been reported so far (reviewed in Engesser & Townsend, 2019; Girard‐Buttoz *et al*., 2022b). This precludes even the possibility that utterances can be used liberally to inform others about a range of synchronous everyday events.

**Fig. 5:**
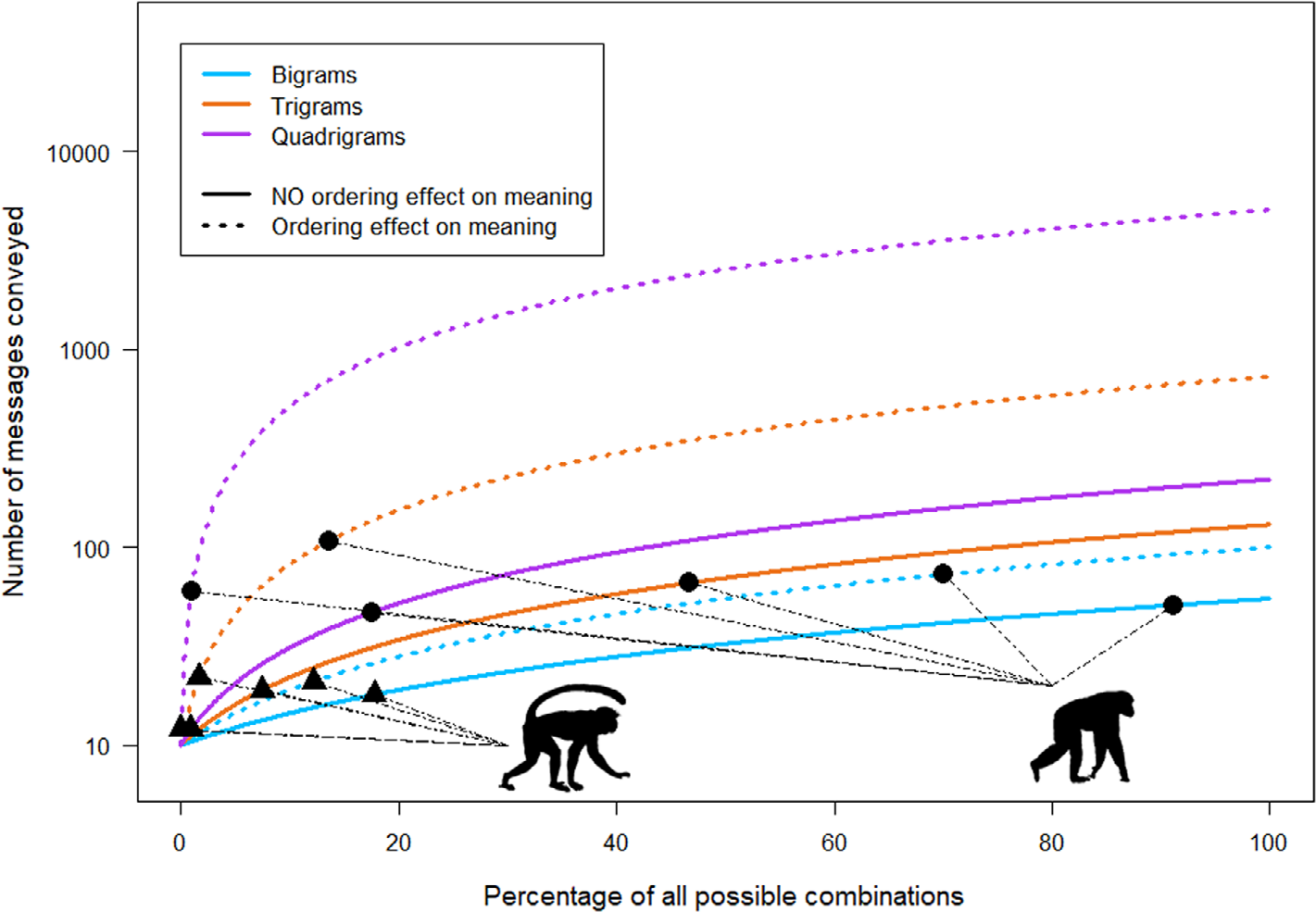
Theoretical sketch illustrating the accumulation of possible messages depending on the number of calls and combinations in a given vocal repertoire. We used empirical vocal data on sooty mangabeys (*Cercocebus atys*) (Sigmundson *et al*., 2025) and chimpanzees (*Pan troglodytes*) (Girard‐Buttoz *et al*., 2022b) to illustrate the number of different bigrams, trigrams and quadrigrams that have been reported so far in each species (with evidence of many more in chimpanzees than mangabeys). Each triangle (mangabey) and circle (chimpanzee) represent the number of combinations reported as a percentage of all possible theoretical bigrams (in blue), trigrams (in orange) and quadrigrams (in purple) that can be formed by recombining all the single calls in their repertoire in all possible orders. These percentages are projected on theoretical lines that represent the total number of messages that could be conveyed if each call combination were to convey a different meaning. Solid line: theoretical number of messages when ordering does not impact meaning, such that A_B and B_A convey the same message. Dashed line: theoretical number of messages if the order in which the calls are combined impacts meaning. The extent to which different vocal sequences convey different meanings remains to be shown for both species. Using not one but several of the combinatorial mechanisms illustrated in Fig. 1 increases the likelihood of reaching this maximum number of messages. In particular, ordering effects dramatically increase message generation potential, especially in longer sequences, although note that structured combinatorial systems such as human language are not expected to combine all sounds in all orders.

However, chimpanzees may liberally use vocal sequences to convey information about concomitant events (Bortolato *et al*., 2023a). Chimpanzees were more likely to emit utterances containing single calls when the signaller encountered only one event at a time (e.g. only feed, greet or aggress). The likelihood of uttering vocal sequences doubled when chimpanzees were engaged in two or more events that occurred concomitantly, such as greeting while feeding (Fig. 3). This indicates not only the structural possibility to liberally combine information within sequences but also a potential to liberally *combine meanings detailing synchronous everyday events* (Bortolato *et al*., 2023a). Indeed, chimpanzees are the first non‐human species to demonstrate the use of not one but all combinatorial mechanisms currently identified in animals (Fig. 1), such that call combinations can either modify, add and generate meanings (Girard‐Buttoz *et al*., 2025) and that meaning is altered by the order of calls in the combination. Furthermore, each mechanism is used for more than one call combination and contextual usage, demonstrating versatility in the usage of combinatorial mechanisms across call combinations. Furthermore, the same call type is used for more than one mechanism. The combined capacity to generate many call combinations across daily life and utilize several combinatorial mechanisms likely exponentially increases the capabilities to generate messages, in particular when the order in which the signals are being combined alters the meaning of the combination (Fig. 5). Similar versatile use of call combinations across contexts has also been reported in wild bonobos (Berthet *et al*., 2025) but the combinatorial mechanisms involved remain debated (see the accompanying eletter by Chemla *et al*. (2025) “The plus and minus of bonobo call combinations”).

The observed versatility in signal combination usage in chimpanzees is unexpected. It demonstrates that a signalling system with mainly inherited single signals, since chimpanzees across populations produce very similar calls (Crockford, 2019), may engage versatility when combining signals. Indeed, signal combinations may show a range of changes in signal usage and function that are not evident for single calls. This demonstrates that the capacity for usage learning, whereby existing signals can be lifted out of one context and used in another, may be critical for generating complex combinatorial communication systems. The extent to which combinatorial flexibility is possible with limited vocal production learning remains to be assessed but may be much more extensive than previously considered possible. Likewise, the extent to which vocal production learning in vocal learning species leads to contextual change also needs to be assessed (see Transition 2 above).

To date, combinatorial mechanisms identified in animal communication mainly refine meanings rather than add meanings together. Indeed, examples of the latter remain rare (Fig. 1). Nonetheless, utterances need to contain more than one meaning in order to express relations between objects and events, a basic function of language. As more combinatorial mechanisms are utilized by a species, the more they achieve versatility in messaging. Thus, we hypothesize that species that use, not one, but various combinatorial mechanisms to expand meanings relating to daily life will be more likely to emit short utterances containing at least two meanings, preluding the emergence of syntax. Whilst Transitions 1–3 may emerge in any and overlapping order, we expect species that have achieved Transition 1 and 2 will be more likely to demonstrate capacities required for Transition 3. Specifically, we expect a certain versatility such that meaning‐bearing calls can be liberally combined with each other to communicate about changing juxtapositions of daily events. We expect versatility in signal usage will require learning processes, possibly including conventionalization.

Once capacities needed for Transitions 1–3 are in place (i.e. once there is flexibility in signal sequence usage, structure, structural rules, and conventionalization of meaning) what constraints might be in place to stop a signalling system that incorporates meaning from rapidly and exponentially expanding? We list three additional considerations that might prevent further expansion.

First, if few signals in the repertoire are under voluntary control, they cannot be liberally combined.

Second, there may be limited numbers of messages to convey, possibly limited by conceptual thought. For example, signals that convey information pertaining to displacement in time and place, have only been reported in one non‐human species, the orangutan (*Pongo abelli*) (Lameira & Call, 2018). In particular, whilst some mammals and birds are capable of planning future actions and have long‐term memories (Baddeley *et al*., 2001; Boeckle & Bugnyar, 2012; Bruck, 2013; Janmaat *et al*., 2014; Keenan *et al*., 2016), they may rarely communicate about past or future events.

Third, there may be limited capacity to encode signals into hierarchical structures using rules that could facilitate meaning extraction from many sequences. The capacity for hierarchically structured thought is considered to constitute a major evolutionary milestone in cognition, impacting both abilities for technological and communicative complexity (Friederici, 2011; Planer, 2023), which we return to in Transition (4).

## VI. TRANSITION 4 – SYNTAX: INTRODUCING LINEAR OR HIERARCHICAL STRUCTURING INTO SIGNAL COMBINATIONS

Human utterances are typically relational, relating different information within an utterance to offer disambiguation, nuance and interpretation about an event or object of interest (Skyrms, 2010; Planer & Sterelny, 2021; Zuberbühler & Bickel, 2022). Whilst relational information minimally requires linear processing (Jackendoff & Wittenberg, 2017), syntactical structures like embedded clauses require hierarchical organization (e.g. Skyrms, 2010; Planer & Sterelny, 2021; Zuberbühler & Bickel, 2022).

Language‐trained apes have demonstrated the use and comprehension of linear symbol constructions in utterances such as ‘Kanzi ball’, referring to self and an item self was interested in having (Truswell, 2017; Berio & Moore, 2023). For decades, such capacities have been considered to be due to enculturation processes derived from human exposure involving exaptation (e.g. Lloyd, 2004). However, recent analyses suggest natural animal communication systems may have similar constructs by combining calls that strongly encode signaller identity together with calls that indicate an event, such as signaller identify + food or greet in chimpanzees (Leroux *et al*., 2021; Girard‐Buttoz *et al*., 2022a). Further, several other compositional‐like vocal utterances have recently been identified in natural *Pan* communication (Berthet *et al*., 2025; Girard‐Buttoz *et al*., 2025), raising the question of whether language‐trained ape skills rather reflect natural communication capacities. Hierarchical structuring of meaning‐bearing units within utterances, on the other hand, is deemed the exclusive realm of human language (Chomsky, 1965; Hauser *et al*., 2002; Fitch & Friederici, 2012; Jackendoff & Wittenberg, 2017; but see Frank, Bod & Christiansen, 2012).

Whilst natural animal communication examples of ordering effects on the meaning of call combinations have been interpreted as encoding syntactical relations, to date, these examples all relate to one or few combinations involving a minority of signals in the repertoire, combining a maximum of two meaning‐bearing units. They detail either short morpho‐syntactical structures, where one unit modifies the meaning of another (Ouattara, Lemasson & Zuberbühler, 2009; Girard‐Buttoz *et al*., 2025) or where the meanings of each constituent call are conjoined (Suzuki *et al*., 2016; Engesser *et al*., 2016; Girard‐Buttoz *et al*., 2025). Such examples are limited to bigrams, offering possible cases of compositionality or proto‐syntax (Suzuki & Zuberbühler, 2019; Planer & Sterelny, 2021; Berthet *et al*., 2022; Schlenker *et al*., 2023). In contrast, hierarchical structuring, which requires recombining and embedding of shorter structures (like bigrams) in longer structures can only appear in combinations containing at least 3 units.

As modelled by Nowak & Krakauer (1999), for a combinatorial system to emerge, first, the total number of messages must likely exceed a critical value well beyond the number of single calls, and second, individual calls should occur in many different messages. For syntax to emerge in communication, we suggest that a critical mass of messages needs to be conveyed with a critical mass of utterances containing at least 3 units.

Once selection pressures are sufficient, a species may invest in the cognitive hardware to generate a MERGE‐like hierarchical system (Bolhuis *et al*., 2014; Rizzi, 2016), enabling recombination or recursion of short chunks (of two meaning‐bearing items) within a phrase, for example embedding the short chunk [the ape] into a longer utterance [I like the ape]. The idea of animals using signal chunks which can be embedded into longer signal sequences evokes such MERGE‐like structures (Zaccarella, Schell & Friederici, 2017; Suzuki & Zuberbühler, 2019; Suzuki & Matsumoto, 2022). Whilst no non‐human species has unequivocally demonstrated the production of relevant meaning‐bearing structures, interestingly, two primate species, chimpanzees and marmosets, demonstrate the capacity to embed independently emitted bigrams into longer vocal sequences (Girard‐Buttoz *et al*., 2022b; Bosshard *et al*., 2024). In chimpanzees, for example, 84% of bigrams (49 out of 58) could have a third call type added to them to produce trigrams.

As well as phrase embedding, another key function of syntax is the denoting of thematic roles of actors and actions, a near universal combinatorial utterance structure in human language (Townsend *et al*., 2018; Zuberbühler & Bickel, 2022). Simple hierarchical structured sentences most commonly contain thematic relationships of agent, action, and patient in various language‐dependent orders. Such sentences typically denote an actor or agent, who does something (action, verb) to someone or something (the patient or object). The syntactical demarcation of the thematic roles means that in each case, agents, actions and patients can be switched out and effortlessly replaced by different agents, actions and patients. This, combined with recursion, facilitates the open‐ended capacity of language to infinitely generate new utterances. It has been argued that the lack of open‐endedness in animal communication in itself indicates a lack of syntax (Bolhuis *et al*., 2018). Palazzolo (2025) alternatively argues for a more continuous evolutionary scenario, that the first hierarchical structures were likely limited in the number of units they could embed.

Many meanings identified as being conveyed by animal signals seem to relate to observable actions (e.g. groom, rest, play, travel, copulate). Some relate to objects (e.g. predator, food), some encode the signaller's identity (e.g. contact calls in dolphins, marmosets, meerkats, elephants, and chimpanzees), the identity of the receiver targeted by the vocalization (in dolphins, King & Janik, 2013; elephants, Pardo *et al*., 2024; and marmosets, Oren *et al*., 2024), or combinations of these (see Fig. 1). Some signal functions also relate to non‐observable actions, where the apparently desired action is not yet visible as the signal precipitates the action (e.g. recruitment signals may precipitate the recruitment of others).

However, roles can only be demarcated in relation to each other, and hence can only begin to become relevant in utterances which contain at least two meanings. Indeed, animal signal combinations which have demonstrated at least two meanings within an utterance are few. Whilst it remains to be determined whether such combinations depict thematic roles in any animal other than humans (see Truswell, 2017; Berio & Moore, 2023), there are several interesting candidates to test. We suggest how these examples may relate to thematic roles. Some may combine action and patient (e.g. Japanese tits: recruit + predator, Suzuki *et al*., 2016; bonobos: recruit + travel, Schamberg *et al*., 2016), or agent and action (e.g. meerkat and banded mongoose: signaller Identity (agent) + action, Jansen *et al*., 2012; Rauber *et al*., 2020; chimpanzee: signaller identity (agent) + greet, Girard‐Buttoz *et al*., 2022a), or signaller identity (agent) + patient (chimpanzee: signaller identity + food, Leroux *et al*., 2021; bonobo: action + signaller identity, Schamberg *et al*., 2016). Particularly the meerkat, mongoose, chimpanzee and bonobo combinations may even point towards animal signal productivity, where a signal retains its meaning or role across combinations (Berthet *et al*., 2022).

Importantly, the semantic category of each signal (agent, action, patient) can emerge without hierarchical syntax simply using a linear grammar. Here, semantic roles can be identified by combining linear ordering of signals and associated pragmatic information (Jackendoff & Wittenberg, 2017; Truswell, 2017; Aussems & Moore, 2025). Such a communication system is not beyond the reach of animals and can be sufficiently generative even for human communication, since some human languages may rely largely on linear grammar (Jackendoff & Wittenberg, 2017). We hypothesise, however, that hierarchical grammar can be seen as a complexification of linear grammar and likely emerged later in evolutionary time, such that hierarchical structures may build on linear structures. Recent studies demonstrate that, conceptually, primates when watching video sequences of animate or inanimate agents and patient interactions effortlessly and appropriately encode agents (actors) and patients (receivers) (Zuberbühler & Bickel, 2022). Taken together, this raises the possibility that vocal sequence structures that combine signals which primarily encode signaller identity with signals that encode actions, like greet, may encode thematic roles like agent + action, such as ‘I greet’, which are also suggested as possible examples of predication (Collier *et al*., 2014; Leroux *et al*., 2021). However, these ideas currently remain untested. Nonetheless, the sense of potential continuity between animal and human communication about events in the real world has given rise to the event perception hypothesis of the evolution of syntax, that the origins of syntax lie in how signallers perceive and mentally represent events (Zuberbühler, 2019, 2020), however tests of this in animal systems are required.

Given this apparent palette of animal signal meaning combinations within utterances, in the search for hierarchical structure in animal utterances, consideration of roles of agent, patient, action, and their productivity, might not be out of reach. Whilst potentially difficult to test using conventional playback experiments, the apparent ease with which chimpanzees assign agency to avatars in virtual worlds (e.g. Allritz *et al*., 2022; Rapport Munro *et al*., 2025b) opens the possibility to test comprehension of conspecific vocal sequences in virtual world contexts. We hypothesize that animals that routinely combine more than two meanings within utterances, recombine signal chunks into longer sequences, and use signals with potential to convey different thematic roles, are prime candidates for utilizing syntax and having generative capacities in utterance construction. Species with Transitions 1–3 would be candidates for examining Transition 4.

## VII. FUTURE DIRECTIONS AND CHALLENGES

Accurate mapping of signal complexity in natural animal systems is relevant across a rich cross‐disciplinary field, from evolutionary models of language evolution (Chomsky, 1965; Nowak & Krakauer, 1999; Nowak & Komarova, 2001; Collier *et al*., 2014; Bolhuis *et al*., 2014; Zuberbühler, 2020; Planer & Sterelny, 2021) to comparative models of gene and brain function (Enard *et al*., 2002; Gallardo *et al*., 2023; Matentzoglu *et al*., 2025; Becker *et al*., 2025), all of which will remain limited until we crack animal signal code to discover its complexity. Natural observations of animals communicating in their natural socio‐ecological environment are an imperative starting point. This determines how and when animals use each signal in their repertoires. More specifically, only natural observation can identify contexts of signal usage and targeted audiences, whether close or distant. Many contexts that trigger signals under natural socio‐ecological conditions are not present in captive settings and cannot be guaranteed in wild settings with human disturbance. For example, chimpanzees emit context‐specific signals in contexts that do not necessarily occur in disturbed habitats or captivity, such as when hunting monkeys or soliciting copulations. In environments with no hunting possibilities or no sexually receptive females, the context‐specific ‘hunt bark’ signal (Mitani & Watts, 1999; Crockford & Boesch, 2003; Mine *et al*., 2022) or palette of copulation solicitation signals (Badihi *et al*., 2023b; Malherbe *et al*., 2025) will be absent in the chimpanzee repertoire. It is unclear how many vocal sequences might only be emitted in wild settings.

Captive settings, however, offer the possibility to assess the impact of exposure to novel contexts on signalling. For example, in captive settings, interactions with humans can elicit novel vocal (African grey parrots, Pepperberg, 2006; orangutan, Lameira *et al*., 2013) and gestural (chimpanzees, Leavens, Hopkins & Bard, 1996; gorilla, Tanner & Perlman, 2017) signals and signal sequences (bonobo, Savage‐Rumbaugh *et al*., 1993; parrot, Pepperberg, 2021), creating new signal forms and functions. The extent to which signals demonstrate new form and function may give insights into the extent to which animal signal repertoires are fixed or flexible. Captive settings also offer a new avenue in deciphering animal sequences with touch‐screen or avatar experiments (Rapport Munro *et al*., 2025a), allowing individuals to actively choose meanings related to particular signals and signal combinations. Hence, a strong approach to decoding animal signal sequences is to compare signal sequence usage across wild and captive populations.

Animal communication research has been effusive, but the capacity to make rapid progress in deciphering animal signal sequences has been limited by highly time‐consuming tools such as field playback experiments or conducting acoustic analyses, which for noisy signals in noisy environments can still require coding elements within sequences by hand. The potential of machine learning to code and classify vocal repertoires into call types assigned to particular contexts will likely have a major impact on progress (Rutz *et al*., 2023). Whilst automated coding and classifying has vastly increased speed of processing and analyses for species with mainly tonal calls in discrete call systems, such as bird song (Xie *et al*., 2023), it remains a significant challenge for graded call systems containing a mix of both tonal and non‐tonal calls, such as is the case for great ape vocal repertoires. At least some progress has been made (Fedurek, Zuberbühler & Dahl, 2016; Arnaud *et al*., 2023; Valet *et al*., 2025) and sizeable vocal recording libraries now exist for training machine learning software (e.g. Girard‐Buttoz *et al*., 2022b; Bortolato *et al*., 2023b).

Surprising, given previous conceptions of fixed, innate vocal repertoires in non‐human primates, is that our closest living relatives are emerging as a front‐runner for vocal communicative complexity. There is versatility in both vocal and gestural sequence construction in the natural communication systems of gorillas, bonobos, and chimpanzees, with at least bonobo and chimpanzee sequences including meaning‐bearing signals (Transition 1). Chimpanzees like humans also demonstrate a dramatic ontogenetic shift across the first decade of life, with infants being rudimentary single signals users and adults relying on combinatorial communication. There is flexibility in signal combination form and function in their natural communication systems, indicating capacity for some social learning of vocal and gestural signals (Transition 2). Bonobos and chimpanzees demonstrate a number of meaning‐bearing call combinations, in which meaning is altered or added compared to the composing units, with chimpanzees and bonobos doing so using several combinatorial mechanisms which flexibility engages different call types (Transition 3). Taken together, great apes demonstrate the most generalized combinatorial capacities, including most of those required to build a complex, combinatorial communication system. Whether these systems indicate a particular shift in the hominid lineage towards becoming versatile, generative communicators requires further research. To date, other species which are good candidates for testing for combinatorial communication systems, because they at least show several signal combinations in different daily life contexts, are also often those that live in more complex societies particularly cooperative or fission–fusion social systems (Figs 1 and 2), evoking the social complexity hypothesis. Nonetheless, to date, identified great ape capacities place them as highly likely candidates to be combinatorial, generative communicators. Given progress in understanding animal signalling relies heavily on observing animals in their natural habitats, and because native great ape habitats are under huge threats, if we are to ever crack the code, both research and conservation efforts are urgent.

## VIII. CONCLUSIONS

(1) Accurate quantitative assessment of the combinatorial capacity of natural animal communication systems is required for comparative and evolutionary models, such as for brain function, genes or language.
(2) Recent animal studies point to a clear empirical direction to decode the combinatorial strategies that animals use in their natural communication. To determine which species are predominantly combinatorial signal users rather than non‐combinatorial signal users, we offer an utterance evolution framework which details four major mechanistic transitions required to build a generative combinatorial system, which may show similarities and differences to language, and which offers quantitative measures for phylogenetic and ontogenetic comparison.
(3) Whilst a wide range of species use signal combinations to modify, add or generate meanings, signal combinations and combinatorial mechanisms are limited per species.
(4) To date, the extant hominids demonstrate the most generalized and versatile combinatorial capacities, including many required to build a combinatorial communication system.
(5) Further research is needed to detail whether the signal combinatorial skills in great ape communication demonstrate that a shift towards language is already evident in our extant hominid relatives, or rather reflects under‐reporting in non‐ape species.
(6) In sum, we argue that recent research showing diverse combinatorial capacities across a wide range of taxa and versatile combinatorial capacities in great apes, means that it is no longer parsimonious to assume that humans are the only species with a generative communication system. Rather, this requires empirical testing.

## Data Availability

Data sharing not applicable to this article as no datasets were generated or analysed during the current study.

## References

[brv70185-bib-0001] Allritz, M. , Call, J. , Schweller, K. , McEwen, E. S. , de Guinea, M. , Janmaat, K. R. L. , Menzel, C. R. & Dolins, F. L. (2022). Chimpanzees (*pan troglodytes*) navigate to find hidden fruit in a virtual environment. Science Advances 8, eabm4754. 35749496 10.1126/sciadv.abm4754 PMC9232100

[brv70185-bib-0002] Amici, F. , Oña, L. & Liebal, K. (2024). Compositionality in primate gestural communication and multicomponent signal displays. International Journal of Primatology 45, 482–500.

[brv70185-bib-0003] Amphaeris, J. , Blumstein, D. T. , Shannon, G. , Tenbrink, T. & Kershenbaum, A. (2023). A multifaceted framework to establish the presence of meaning in non‐human communication. Biological Reviews 98, 1887–1909. 37340613 10.1111/brv.12989

[brv70185-bib-0004] Anikin, A. , Canessa‐Pollard, V. , Pisanski, K. , Massenet, M. & Reby, D. (2023). Beyond speech: exploring diversity in the human voice. iScience 26, 108204. 37908309 10.1016/j.isci.2023.108204 PMC10613903

[brv70185-bib-0005] Arnaud, V. , Pellegrino, F. , Keenan, S. , St‐Gelais, X. , Mathevon, N. , Levréro, F. & Coupé, C. (2023). Improving the workflow to crack small, unbalanced, Noisy, but genuine (SUNG) datasets in bioacoustics: the case of bonobo calls. PLoS Computational Biology 19, e1010325. 37053268 10.1371/journal.pcbi.1010325 PMC10129004

[brv70185-bib-0006] Arnold, K. & Zuberbühler, K. (2006). Semantic combinations in primate calls. Nature 441, 303. 16710411 10.1038/441303a

[brv70185-bib-0007] Arnold, K. & Zuberbühler, K. (2008). Meaningful call combinations in a non‐human primate. Current Biology 18, R202–R203. 18334192 10.1016/j.cub.2008.01.040

[brv70185-bib-0008] Aussems, S. & Moore, R. (2025). Signal use and pragmatics in the first natural language users. In Evolutionary Pragmatics: Communicative Interaction and the Origins of Language (Volume 21), p. 30. Oxford University Press, Oxford, UK.

[brv70185-bib-0009] Baddeley, A. , Conway, M. , Aggleton, J. , Clayton, N. S. , Griffiths, D. P. , Emery, N. J. & Dickinson, A. (2001). Elements of episodic–like memory in animals. Philosophical Transactions of the Royal Society of London. Series B: Biological Sciences 356, 1483–1491. 11571038 10.1098/rstb.2001.0947 PMC1088530

[brv70185-bib-0010] Badihi, G. , Graham, K. E. , Fallon, B. , Safryghin, A. , Soldati, A. , Zuberbühler, K. & Hobaiter, C. (2023). Dialects in leaf‐clipping and other leaf‐modifying gestures between neighbouring communities of east African chimpanzees. Scientific Reports 13, 147. 36604445 10.1038/s41598-022-25814-x PMC9814361

[brv70185-bib-0011] Becker, Y. , Eichner, C. , Paquette, M. , Bock, C. , Girard‐Buttoz, C. , Jäger, C. , Gräßle, T. , Deschner, T. , Gunz, P. , Wittig, R. M. , Crockford, C. , Friederici, A. D. & Anwander, A. (2025). Long arcuate fascicle in wild and captive chimpanzees as a potential structural precursor of the language network. Nature Communications 16, 4485. 10.1038/s41467-025-59254-8 PMC12081605 40374618

[brv70185-bib-0012] Bergman, T. J. , Beehner, J. C. , Cheney, D. L. & Seyfarth, R. M. (2003). Hierarchical classification by rank and kinship in baboons. Science 302, 1234–1236. 14615544 10.1126/science.1087513

[brv70185-bib-0013] Berio, L. & Moore, R. (2023). Great ape enculturation studies: a neglected resource in cognitive development research. Biology & Philosophy 38, 17.

[brv70185-bib-0014] Berthet, M. , Coye, C. , Dezecache, G. & Kuhn, J. (2022). Animal linguistics: a primer. Biological Reviews 98(1), 81–98. 36189714 10.1111/brv.12897 PMC10091714

[brv70185-bib-0015] Berthet, M. , Mesbahi, G. , Pajot, A. , Cäsar, C. , Neumann, C. & Zuberbühler, K. (2019). Titi monkeys combine alarm calls to create probabilistic meaning. Science Advances 5, eaav3991. 31123704 10.1126/sciadv.aav3991 PMC6527438

[brv70185-bib-0016] Berthet, M. , Surbeck, M. & Townsend, S. W. (2025). Extensive compositionality in the vocal system of bonobos. Science 388, 104–108. 40179186 10.1126/science.adv1170

[brv70185-bib-0017] Berwick, R. C. , Okanoya, K. , Beckers, G. J. L. & Bolhuis, J. J. (2011). Songs to syntax: the linguistics of birdsong. Trends in Cognitive Sciences 15, 113–121. 21296608 10.1016/j.tics.2011.01.002

[brv70185-bib-0018] Boë, L.‐J. , Berthommier, F. , Legou, T. , Captier, G. , Kemp, C. , Sawallis, T. R. , Becker, Y. , Rey, A. & Fagot, J. (2017). Evidence of a vocalic proto‐system in the baboon (*Papio papio*) suggests pre‐hominin speech precursors. PLoS One 12, e0169321. 28076426 10.1371/journal.pone.0169321 PMC5226677

[brv70185-bib-0019] Boeckle, M. & Bugnyar, T. (2012). Long‐term memory for affiliates in ravens. Current Biology 22, 801–806. 22521788 10.1016/j.cub.2012.03.023 PMC3348500

[brv70185-bib-0020] Bolhuis, J. J. , Beckers, G. J. L. , Huybregts, M. A. C. , Berwick, R. C. & Everaert, M. B. H. (2018). The slings and arrows of comparative linguistics. PLoS Biology 16, e3000019. 30248090 10.1371/journal.pbio.3000019 PMC6152852

[brv70185-bib-0021] Bolhuis, J. J. , Tattersall, I. , Chomsky, N. & Berwick, R. C. (2014). How could language have evolved? PLoS Biology 12, e1001934. 25157536 10.1371/journal.pbio.1001934 PMC4144795

[brv70185-bib-0022] Bortolato, T. , Friederici, A. D. , Girard‐Buttoz, C. , Wittig, R. M. & Crockford, C. (2023 *a*). Chimpanzees show the capacity to communicate about concomitant daily life events. iscience 26, 108090. 37876805 10.1016/j.isci.2023.108090 PMC10590744

[brv70185-bib-0023] Bortolato, T. , Mundry, R. , Wittig, R. M. , Girard‐Buttoz, C. & Crockford, C. (2023 *b*). Slow development of vocal sequences through ontogeny in wild chimpanzees (*Pan troglodytes verus*). Developmental Science 26, e13350. 36440660 10.1111/desc.13350

[brv70185-bib-0024] Bosshard, A. B. , Burkart, J. M. , Merlo, P. , Cathcart, C. , Townsend, S. W. & Bickel, B. (2024). Beyond bigrams: call sequencing in the common marmoset (*Callithrix jacchus*) vocal system. Royal Society Open Science 11, 240218. 39507993 10.1098/rsos.240218 PMC11537759

[brv70185-bib-0025] Bouchard, A. & Zuberbühler, K. (2022). An intentional cohesion call in male chimpanzees of Budongo Forest. Animal Cognition 25, 853–866. 35044524 10.1007/s10071-022-01597-6 PMC9334450

[brv70185-bib-0026] Bouchet, H. , Blois‐Heulin, C. & Lemasson, A. (2013). Social complexity parallels vocal complexity: a comparison of three non‐human primate species. Frontiers in Psychology 4, 1–15. 23847565 10.3389/fpsyg.2013.00390 PMC3705190

[brv70185-bib-0027] Bradbury, J. W. & Balsby, T. J. S. (2016). The functions of vocal learning in parrots. Behavioral Ecology and Sociobiology 70, 293–312.

[brv70185-bib-0028] Bruck, J. N. (2013). Decades‐long social memory in bottlenose dolphins. Proceedings of the Royal Society B‐Biological Sciences 280, UNSP 20131726. 10.1098/rspb.2013.1726 PMC3757989 23926160

[brv70185-bib-0029] Catchpole, C. K. & Slater, P. J. B. (2003). Bird Song: Biological Themes and Variations. Cambridge University Press, Cambridge, UK.

[brv70185-bib-0030] Chemla, E. , Brochhagen, T. & Steinert‐Threlkeld, S. (2025). The plus and minus of bonobo call combinations. Science 388, 104–108.40179186

[brv70185-bib-0031] Cheney, D. L. & Seyfarth, R. M. (1990). Attending to behaviour versus attending to knowledge: examining monkeys' attribution of mental states. Animal Behaviour 40, 742–753.

[brv70185-bib-0032] Cheney, D. L. & Seyfarth, R. M. (2018). Flexible usage and social function in primate vocalizations. Proceedings of the National Academy of Sciences 115, 1974–1979. 10.1073/pnas.1717572115 PMC5834704 29432157

[brv70185-bib-0033] Chomsky, N. (1965). Aspects of the Theory of Syntax. MIT Press, Cambridge, MA, USA.

[brv70185-bib-0034] Collier, K. , Bickel, B. , van Schaik, C. P. , Manser, M. B. & Townsend, S. W. (2014). Language evolution: syntax before phonology? Proceedings of the Royal Society B: Biological Sciences 281, 20140263. 10.1098/rspb.2014.0263 PMC4083781 24943364

[brv70185-bib-0035] Coye, C. , Ouattara, K. , Zuberbühler, K. & Lemasson, A. (2015). Suffixation influences receivers' behaviour in non‐human primates. Proceedings of the Royal Society B: Biological Sciences 282, 20150265. 10.1098/rspb.2015.0265 PMC4424650 25925101

[brv70185-bib-0036] Coye, C. , Zuberbühler, K. & Lemasson, A. (2016). Morphologically structured vocalizations in female Diana monkeys. Animal Behaviour 115, 97–105.

[brv70185-bib-0037] Crockford, C. (2019). Why does the chimpanzee vocal repertoire remain poorly understood? – and what can be done about it. In The Chimpanzees of the Taï Forest: 40 Years of Research (eds C. Boesch , R. M. Wittig , C. Crockford , L. Vigilant , T. Deschner and F. Leendertz ), pp. 394–409. Cambridge University Press, Cambridge, UK.

[brv70185-bib-0038] Crockford, C. & Boesch, C. (2003). Context‐specific calls in wild chimpanzees, *pan troglodytes verus*: analysis of barks. Animal Behaviour 66, 115–125.

[brv70185-bib-0039] Crockford, C. , Herbinger, I. , Vigilant, L. & Boesch, C. (2004). Wild chimpanzees produce group‐specific calls: a case for vocal learning? Ethology 110, 221–243.

[brv70185-bib-0040] Crockford, C. , Wittig, R. M. , Mundry, R. & Zuberbühler, K. (2012). Wild chimpanzees inform ignorant group members of danger. Current Biology 22, 142–146. 22209531 10.1016/j.cub.2011.11.053

[brv70185-bib-0041] Crockford, C. , Wittig, R. M. , Seyfarth, R. M. & Cheney, D. L. (2007). Baboons eavesdrop to deduce mating opportunities. Animal Behaviour 73, 885–890.

[brv70185-bib-0042] Crockford, C. , Wittig, R. M. & Zuberbühler, K. (2017). Vocalizing in chimpanzees is influenced by social‐cognitive processes. Science Advances 3, e1701742. 29152569 10.1126/sciadv.1701742 PMC5687857

[brv70185-bib-0043] Dingemanse, M. , Blasi, D. E. , Lupyan, G. , Christiansen, M. H. & Monaghan, P. (2015). Arbitrariness, iconicity, and systematicity in language. Trends in Cognitive Sciences 19, 603–615. 26412098 10.1016/j.tics.2015.07.013

[brv70185-bib-0044] Dutour, M. , Lengagne, T. & Léna, J.‐P. (2019). Syntax manipulation changes perception of mobbing call sequences across passerine species. Ethology 125, 635–644.

[brv70185-bib-0045] Elemans, C. P. H. , Mead, A. F. , Rome, L. C. & Goller, F. (2008). Superfast vocal muscles control song production in songbirds. PLoS One 3, e2581. 18612467 10.1371/journal.pone.0002581 PMC2440420

[brv70185-bib-0046] Elie, J. E. , de Witasse‐Thézy, A. , Thomas, L. , Malit, B. & Theunissen, F. E. (2024). Categorical and semantic perception of the meaning of call‐types in zebra finches. Science 389(6766), 1210–1215. 10.1126/science.ads8482 PMC12721838 40966354

[brv70185-bib-0047] Enard, W. , Przeworski, M. , Fisher, S. E. , Lai, C. S. L. , Wiebe, V. , Kitano, T. , Monaco, A. P. & Pääbo, S. (2002). Molecular evolution of FOXP2, a gene involved in speech and language. Nature 418, 869–872. 12192408 10.1038/nature01025

[brv70185-bib-0048] Engesser, S. , Ridley, A. R. & Townsend, S. W. (2016). Meaningful call combinations and compositional processing in the southern pied babbler. Proceedings of the National Academy of Sciences 113, 5976–5981. 10.1073/pnas.1600970113 PMC4889383 27155011

[brv70185-bib-0049] Engesser, S. & Townsend, S. W. (2019). Combinatoriality in the vocal systems of nonhuman animals. WIREs Cognitive Science 10, e1493. 30724476 10.1002/wcs.1493

[brv70185-bib-0050] Fedorenko, E. , Piantadosi, S. T. & Gibson, E. A. F. (2024). Language is primarily a tool for communication rather than thought. Nature 630, 575–586. 38898296 10.1038/s41586-024-07522-w

[brv70185-bib-0051] Fedurek, P. & Slocombe, K. E. (2011). Primate vocal communication: a useful tool for understanding human speech and language evolution? Human Biology 83, 153–173. 21615284 10.3378/027.083.0202

[brv70185-bib-0052] Fedurek, P. , Zuberbühler, K. & Dahl, C. D. (2016). Sequential information in a great ape utterance. Scientific Reports 6, 38226. 27910886 10.1038/srep38226 PMC5133612

[brv70185-bib-0053] Fischer, J. (1998). Barbary macaques categorize shrill barks into two call types. Animal Behaviour 55, 799–807. 9632468 10.1006/anbe.1997.0663

[brv70185-bib-0054] Fischer, J. & Hammerschmidt, K. (2020). Towards a new taxonomy of primate vocal production learning. Philosophical Transactions of the Royal Society, B: Biological Sciences 375, 20190045. 10.1098/rstb.2019.0045 PMC6895554 31735147

[brv70185-bib-0055] Fischer, J. & Price, T. (2017). Meaning, intention, and inference in primate vocal communication. Neuroscience & Biobehavioral Reviews 82, 22–31. 27773691 10.1016/j.neubiorev.2016.10.014

[brv70185-bib-0056] Fitch, W. T. & Friederici, A. D. (2012). Artificial grammar learning meets formal language theory: an overview. Philosophical Transactions of the Royal Society, B: Biological Sciences 367, 1933–1955. 10.1098/rstb.2012.0103 PMC3367694 22688631

[brv70185-bib-0057] Fitch, W. T. , Huber, L. & Bugnyar, T. (2010). Social cognition and the evolution of language: constructing cognitive phylogenies. Neuron 65, 795–814. 20346756 10.1016/j.neuron.2010.03.011 PMC4415479

[brv70185-bib-0058] Frank, S. L. , Bod, R. & Christiansen, M. H. (2012). How hierarchical is language use? Proceedings of the Royal Society B: Biological Sciences 279, 4522–4531. 10.1098/rspb.2012.1741 PMC3479729 22977157

[brv70185-bib-0059] Franke, M. (2016). The evolution of compositionality in signaling games. Journal of Logic, Language and Information 25, 355–377.

[brv70185-bib-0060] Freeberg, T. M. (2008). Complexity in the chick‐a‐dcall of Carolina chickadees (*Poecile Carolinensis*): associations of context and signaler behavior to call structure. The Auk 125, 896–907.

[brv70185-bib-0061] Freeberg, T. M. , Dunbar, R. I. M. & Ord, T. J. (2012). Social complexity as a proximate and ultimate factor in communicative complexity. Philosophical Transactions of the Royal Society, B: Biological Sciences 367, 1785–1801. 10.1098/rstb.2011.0213 PMC3367695 22641818

[brv70185-bib-0062] Friederici, A. D. (2011). The brain basis of language processing: from structure to function. Physiological Reviews 91, 1357–1392. 22013214 10.1152/physrev.00006.2011

[brv70185-bib-0063] Gallardo, G. , Eichner, C. , Sherwood, C. C. , Hopkins, W. D. , Anwander, A. & Friederici, A. D. (2023). Morphological evolution of language‐relevant brain areas. PLoS Biology 21, e3002266. 37656748 10.1371/journal.pbio.3002266 PMC10501646

[brv70185-bib-0064] Gallot, Q. , Tillé, Y. , Depriester, C. , Moran, S. & Zuberbühler, K. (2025). A primate grammar enabling incremental processing. iScience 28(4), 112229. 40241766 10.1016/j.isci.2025.112229 PMC12003008

[brv70185-bib-0065] Gasparri, L. , Filippi, P. , Wild, M. & Glock, H.‐J. (2023). Notions of arbitrariness. Mind & Language 38, 1120–1137.

[brv70185-bib-0066] Genty, E. & Zuberbühler, K. (2014). Spatial reference in a bonobo gesture. Current Biology 24, 1601–1605. 24998531 10.1016/j.cub.2014.05.065

[brv70185-bib-0067] Girard‐Buttoz, C. , Bortolato, T. , Laporte, M. , Grampp, M. , Zuberbühler, K. , Wittig, R. M. & Crockford, C. (2022 *a*). Population‐specific call order in chimpanzee greeting vocal sequences. iScience 25, 104851. 36034222 10.1016/j.isci.2022.104851 PMC9399282

[brv70185-bib-0068] Girard‐Buttoz, C. , Neumann, C. , Bortolato, T. , Zaccarella, E. , Friederici, A. D. , Wittig, R. M. & Crockford, C. (2025). Versatile use of chimpanzee call combinations promotes meaning expansion. Science Advances 11, eadq2879. 40344055 10.1126/sciadv.adq2879 PMC12063654

[brv70185-bib-0069] Girard‐Buttoz, C. , Surbeck, M. , Samuni, L. , Tkaczynski, P. , Boesch, C. , Fruth, B. , Wittig, R. M. , Hohmann, G. & Crockford, C. (2020). Information transfer efficiency differs in wild chimpanzees and bonobos, but not social cognition. Proceedings of the Royal Society B: Biological Sciences 287, 20200523. 10.1098/rspb.2020.0523 PMC7329035 32576115

[brv70185-bib-0070] Girard‐Buttoz, C. , Zaccarella, E. , Bortolato, T. , Friederici, A. D. , Wittig, R. M. & Crockford, C. (2022 *b*). Chimpanzees produce diverse vocal sequences with ordered and recombinatorial properties. Communications Biology 5, 1–15. 35577891 10.1038/s42003-022-03350-8 PMC9110424

[brv70185-bib-0071] Graham, K. E. , Furuichi, T. & Byrne, R. W. (2020). Context, not sequence order, affects the meaning of bonobo (*Pan paniscus*) gestures. Gesture 19, 335–364.

[brv70185-bib-0072] Graham, K. E. , Wilke, C. , Lahiff, N. J. & Slocombe, K. E. (2019). Scratching beneath the surface: intentionality in great ape signal production. Philosophical Transactions of the Royal Society, B: Biological Sciences 375, 20180403. 10.1098/rstb.2018.0403 PMC6895546 31735155

[brv70185-bib-0073] Grampp, M. , Samuni, L. , Girard‐Buttoz, C. , León, J. , Zuberbühler, K. , Tkaczynski, P. , Wittig, R. M. & Crockford, C. (2023). Social uncertainty promotes signal complexity during approaches in wild chimpanzees (*Pan troglodytes verus*) and mangabeys (*Cercocebus atys atys*). Royal Society Open Science 10, 231073. 38034119 10.1098/rsos.231073 PMC10685125

[brv70185-bib-0074] Gultekin, Y. B. , Hildebrand, D. G. C. , Hammerschmidt, K. & Hage, S. R. (2021). High plasticity in marmoset monkey vocal development from infancy to adulthood. Science Advances 7, eabf2938. 34193413 10.1126/sciadv.abf2938 PMC8245035

[brv70185-bib-0075] Hammerschmidt, K. & Fischer, J. (2019). Baboon vocal repertoires and the evolution of primate vocal diversity. Journal of Human Evolution 126, 1–13. 30583838 10.1016/j.jhevol.2018.10.010

[brv70185-bib-0076] Hauser, M. D. , Chomsky, N. & Fitch, W. T. (2002). The Faculty of Language: what is it, who has it, and how did it evolve? Science 298, 1569–1579. 12446899 10.1126/science.298.5598.1569

[brv70185-bib-0077] Hedwig, D. , Hammerschmidt, K. , Mundry, R. , Robbins, M. M. & Boesch, C. (2014). Acoustic structure and variation in mountain and western gorilla close calls: a syntactic approach. Behaviour 151(8), 1091–1120.

[brv70185-bib-0078] Hedwig, D. & Kohlberg, A. (2024). Call combination in African forest elephants *Loxodonta cyclotis* . PLoS One 19, e0299656. 38498501 10.1371/journal.pone.0299656 PMC10947659

[brv70185-bib-0079] Heintz, C. & Scott‐Phillips, T. (2023). Expression unleashed: the evolutionary and cognitive foundations of human communication. Behavioral and Brain Sciences 46, e1. 10.1017/S0140525X22000012 34983701

[brv70185-bib-0080] Hobaiter, C. & Byrne, R. W. (2011). Serial gesturing by wild chimpanzees: its nature and function for communication. Animal Cognition 14, 827–838. 21562816 10.1007/s10071-011-0416-3

[brv70185-bib-0081] Hockett, C. F. (1959). Animal ‘languages’ and human language. Human Biology 31, 32–39. 13640650

[brv70185-bib-0082] Jackendoff, R. & Wittenberg, E. (2017). Linear grammar as a possible stepping‐stone in the evolution of language. Psychonomic Bulletin & Review 24, 219–224. 27368633 10.3758/s13423-016-1073-y

[brv70185-bib-0083] Janik, V. M. & Slater, P. J. B. (2000). The different roles of social learning in vocal communication. Animal Behaviour 60, 1–11. 10924198 10.1006/anbe.2000.1410

[brv70185-bib-0084] Janmaat, K. R. L. , Polansky, L. , Ban, S. D. & Boesch, C. (2014). Wild chimpanzees plan their breakfast time, type, and location. Proceedings of the National Academy of Sciences 111, 16343–16348. 10.1073/pnas.1407524111 PMC4246305 25349399

[brv70185-bib-0085] Jansen, D. A. , Cant, M. A. & Manser, M. B. (2012). Segmental concatenation of individual signatures and context cues in banded mongoose (*Mungos mungo*) close calls. BMC Biology 10, 1–11. 23206242 10.1186/1741-7007-10-97 PMC3529192

[brv70185-bib-0086] Jarvis, E. D. (2019). Evolution of vocal learning and spoken language. Science 366, 50–54. 31604300 10.1126/science.aax0287

[brv70185-bib-0087] Jolly, A. (1966). Lemur social behavior and primate intelligence: step from prosimian to monkey intelligence probably took place in a social context. Science 153, 501. 5938775 10.1126/science.153.3735.501

[brv70185-bib-0088] Kalan, A. K. , Nakano, R. & Warshawski, L. (2025). What we know and don't know about great ape cultural communication in the wild. American Journal of Primatology 87, e23560. 37828822 10.1002/ajp.23560 PMC11650962

[brv70185-bib-0089] Kappeler, P. M. (2019). A framework for studying social complexity. Behavioral Ecology and Sociobiology 73, 13.

[brv70185-bib-0090] Keenan, S. , Mathevon, N. , Stevens, J. M. , Guéry, J. P. , Zuberbühler, K. & Levréro, F. (2016). Enduring voice recognition in bonobos. Scientific Reports 6, 22046. 26911199 10.1038/srep22046 PMC4766561

[brv70185-bib-0091] King, S. L. & Janik, V. M. (2013). Bottlenose dolphins can use learned vocal labels to address each other. Proceedings of the National Academy of Sciences 110, 13216–13221. 10.1073/pnas.1304459110 PMC3740840 23878217

[brv70185-bib-0092] Laidre, M. E. & Johnstone, R. A. (2013). Animal signals. Current Biology 23, R829–R833. 24070440 10.1016/j.cub.2013.07.070

[brv70185-bib-0093] Lameira, A. R. & Call, J. (2018). Time–space–displaced responses in the orangutan vocal system. Science Advances 4, eaau3401. 30443595 10.1126/sciadv.aau3401 PMC6235548

[brv70185-bib-0094] Lameira, A. R. , Hardus, M. E. , Kowalsky, B. , de Vries, H. , Spruijt, B. M. , Sterck, E. H. M. , Shumaker, R. W. & Wich, S. A. (2013). Orangutan (*Pongo spp*.) whistling and implications for the emergence of an open‐ended call repertoire: a replication and extension. The Journal of the Acoustical Society of America 134, 2326–2335. 23967963 10.1121/1.4817929

[brv70185-bib-0095] Le Floch, A. , Girard‐Buttoz, C. , Azaiez, T. S. , Bande, N. , Wittig, R. M. , Moran, S. , Zuberbühler, K. & Crockford, C. (2026 *a*). Rule‐based sequences in sooty mangabey vocal communication. Royal Society Open Science 13, 251944.

[brv70185-bib-0096] Le Floch, A. , Girard‐Buttoz, C. , Neumann, C. , Azaiez, T. S. , Bande, N. , Wittig, R. M. , Zuberbühler, K. & Crockford, C. (2026 *b*). Call combination order and iterations may shift meaning in sooty mangabey vocal sequences. BMC Biology 24, 81. 41721371 10.1186/s12915-026-02528-4 PMC13032478

[brv70185-bib-0097] Leavens, D. A. , Hopkins, W. D. & Bard, K. A. (1996). Indexical and referential pointing in chimpanzees (*Pan troglodytes*). Journal of Comparative Psychology 110, 346–353. 8956506 10.1037/0735-7036.110.4.346 PMC2175394

[brv70185-bib-0098] León, J. , Thiriau, C. , Crockford, C. & Zuberbühler, K. (2023). Comprehension of own and other species' alarm calls in sooty mangabey vocal development. Behavioral Ecology and Sociobiology 77, 1–17. 10.1007/s00265-023-03318-6 PMC10205891 37234238

[brv70185-bib-0099] Leroux, M. , Bosshard, A. B. , Chandia, B. , Manser, A. , Zuberbühler, K. & Townsend, S. W. (2021). Chimpanzees combine pant hoots with food calls into larger structures. Animal Behaviour 179, 41–50.

[brv70185-bib-0100] Leroux, M. , Schel, A. M. , Wilke, C. , Chandia, B. , Zuberbühler, K. , Slocombe, K. E. & Townsend, S. W. (2023). Call combinations and compositional processing in wild chimpanzees. Nature Communications 14, 2225. 10.1038/s41467-023-37816-y PMC10160036 37142584

[brv70185-bib-0101] Lewis, D. (1969 *a*). Convention: A Philosophical Study. Havard University Press, Cambridge, MA, USA.

[brv70185-bib-0102] Lewis, D. (1969 *b*). Convention. Blackwell, Oxford.

[brv70185-bib-0103] Lloyd, E. A. (2004). Kanzi, evolution, and language. Biology and Philosophy 19(4), 577–588.

[brv70185-bib-0104] Loning, H. , Griffith, S. C. & Naguib, M. (2024). The ecology of zebra finch song and its implications for vocal communication in multi‐level societies. Philosophical Transactions of the Royal Society, B: Biological Sciences 379, 20230191. 10.1098/rstb.2023.0191 PMC11391294 38768203

[brv70185-bib-0105] Maciej, P. , Ndao, I. , Hammerschmidt, K. & Fischer, J. (2013). Vocal communication in a complex multi‐level society: constrained acoustic structure and flexible call usage in Guinea baboons. Frontiers in Zoology 10, 1–15. 24059742 10.1186/1742-9994-10-58 PMC3849383

[brv70185-bib-0106] Malherbe, M. , Kpazahi, H. N. , Kone, I. , Samuni, L. , Crockford, C. & Wittig, R. M. (2025). Signal traditions and cultural loss in chimpanzees. Current Biology 35, R87–R88. 39904312 10.1016/j.cub.2024.12.008

[brv70185-bib-0107] Manser, M. B. , Jansen, D. A. W. A. M. , Graw, B. , Hollén, L. I. , Bousquet, C. A. H. , Furrer, R. D. & le Roux, A. (2014). Chapter six – vocal complexity in meerkats and other mongoose species. In Advances in the Study of Behavior (eds M. Naguib , L. Barrett , H. J. Brockmann , S. Healy , J. C. Mitani , T. J. Roper and L. W. Simmons ), pp. 281–310. Academic Press, Cambridge, MA, USA.

[brv70185-bib-0108] Marshall, A. J. , Wrangham, R. W. & Arcadi, A. C. (1999). Does learning affect the structure of vocalizations in chimpanzees? Animal Behaviour 58, 825–830. 10512656 10.1006/anbe.1999.1219

[brv70185-bib-0109] Matentzoglu, N. , Bello, S. M. , Stefancsik, R. , Alghamdi, S. M. , Anagnostopoulos, A. V. , Balhoff, J. P. , Balk, M. A. , Bradford, Y. M. , Bridges, Y. , Callahan, T. J. , Caufield, H. , Cuzick, A. , Carmody, L. C. , Caron, A. R. , de Souza, V. , *et al*. (2025). The unified phenotype ontology: a framework for cross‐species integrative phenomics. Genetics 229, iyaf027. 40048704 10.1093/genetics/iyaf027 PMC11912833

[brv70185-bib-0110] McComb, K. & Semple, S. (2005). Coevolution of vocal communication and sociality in primates. Biology Letters 1, 381–385. 17148212 10.1098/rsbl.2005.0366 PMC1626386

[brv70185-bib-0111] Mine, J. G. , Slocombe, K. E. , Willems, E. P. , Gilby, I. C. , Yu, M. , Thompson, M. E. , Muller, M. N. , Wrangham, R. W. , Townsend, S. W. & Machanda, Z. P. (2022). Vocal signals facilitate cooperative hunting in wild chimpanzees. *Science* . Advances 8, eabo5553. 10.1126/sciadv.abo5553 PMC9337754 35905190

[brv70185-bib-0112] Mitani, J. C. , Hunley, K. L. & Murdoch, M. E. (1999). Geographic variation in the calls of wild chimpanzees: a reassessment. American Journal of Primatology 47, 133–151. 9973267 10.1002/(SICI)1098-2345(1999)47:2<133::AID-AJP4>3.0.CO;2-I

[brv70185-bib-0113] Mitani, J. C. & Nishida, T. (1993). Contexts and social correlates of long‐distance calling by male chimpanzees. Animal Behaviour 45, 735–746.

[brv70185-bib-0114] Mitani, J. C. & Watts, D. P. (1999). Demographic influences on the hunting behavior of chimpanzees. American Journal of Physical Anthropology 109, 439–454. 10423261 10.1002/(SICI)1096-8644(199908)109:4<439::AID-AJPA2>3.0.CO;2-3

[brv70185-bib-0115] Moore, R. (2013). Imitation and conventional communication. Biology & Philosophy 28, 481–500.

[brv70185-bib-0116] Moore, R. (2016). Meaning and ostension in great ape gestural communication. Animal Cognition 19, 223–231. 26223212 10.1007/s10071-015-0905-x

[brv70185-bib-0117] Moran, S. , McCloy, D. & Wright, R. (2012). Revisiting population size vs. phoneme inventory size. Language 88, 877–893.

[brv70185-bib-0118] Nowak, M. A. & Komarova, N. L. (2001). Towards an evolutionary theory of language. Trends in Cognitive Sciences 5, 288–295. 11425617 10.1016/s1364-6613(00)01683-1

[brv70185-bib-0119] Nowak, M. A. & Krakauer, D. C. (1999). The evolution of language. Proceedings of the National Academy of Sciences 96, 8028–8033. 10.1073/pnas.96.14.8028 PMC22182 10393942

[brv70185-bib-0120] Nowak, M. A. , Plotkin, J. B. & Jansen, V. A. A. (2000). The evolution of syntactic communication. Nature 404, 495–498. 10761917 10.1038/35006635

[brv70185-bib-0121] Nowicki, S. & Searcy, W. A. (2014). The evolution of vocal learning. Current Opinion in Neurobiology 28, 48–53. 25033109 10.1016/j.conb.2014.06.007

[brv70185-bib-0122] Oren, G. , Shapira, A. , Lifshitz, R. , Vinepinsky, E. , Cohen, R. , Fried, T. , Hadad, G. P. & Omer, D. (2024). Vocal labeling of others by nonhuman primates. Science 385, 996–1003. 39208084 10.1126/science.adp3757

[brv70185-bib-0123] Ouattara, K. , Lemasson, A. & Zuberbühler, K. (2009). Campbell's monkeys use affixation to alter call meaning. PLoS One 4, e7808. 19915663 10.1371/journal.pone.0007808 PMC2771905

[brv70185-bib-0124] Owren, M. J. , Dieter, J. A. , Seyfarth, R. M. & Cheney, D. L. (1993). Vocalizations of rhesus (*Macaca mulatta*) and Japanese (*M. Fuscata*) macaques cross‐fostered between species show evidence of only limited modification. Developmental Psychobiology 26, 389–406. 8270122 10.1002/dev.420260703

[brv70185-bib-0125] Palazzolo, G. (2025). A bounded hierarchy framework for the evolution of syntax. Biology & Philosophy 40, 26. 41098947 10.1007/s10539-025-09998-w PMC12518414

[brv70185-bib-0126] Pardo, M. A. , Fristrup, K. , Lolchuragi, D. S. , Poole, J. H. , Granli, P. , Moss, C. , Douglas‐Hamilton, I. & Wittemyer, G. (2024). African elephants address one another with individually specific name‐like calls. Nature Ecology & Evolution 8, 1353–1364. 38858512 10.1038/s41559-024-02420-w

[brv70185-bib-0127] Pardo, M. A. , Poole, J. H. , Stoeger, A. S. , Wrege, P. H. , O'Connell‐Rodwell, C. E. , Padmalal, U. K. & de Silva, S. (2019). Differences in combinatorial calls among the 3 elephant species cannot be explained by phylogeny. Behavioral Ecology 30, 809–820.

[brv70185-bib-0128] Peckre, L. , Kappeler, P. M. & Fichtel, C. (2019). Clarifying and expanding the social complexity hypothesis for communicative complexity. Behavioral Ecology and Sociobiology 73, 11.

[brv70185-bib-0129] Pepperberg, I. M. (2006). Cognitive and communicative abilities of Grey parrots. Applied Animal Behaviour Science 100, 77–86.

[brv70185-bib-0130] Pepperberg, I. M. (2021). Nonhuman and nonhuman‐human communication: some issues and questions. Frontiers in Psychology 12, 647841. 34630194 10.3389/fpsyg.2021.647841 PMC8495326

[brv70185-bib-0131] Petkov, C. I. & Jarvis, E. (2012). Birds, primates, and spoken language origins: behavioral phenotypes and neurobiological substrates. Frontiers in Evolutionary Neuroscience 4, 12. 22912615 10.3389/fnevo.2012.00012 PMC3419981

[brv70185-bib-0132] Planer, R. & Sterelny, K. (2021). From Signal to Symbol: The Evolution of Language. MIT Press, Cambridge, MA, USA.

[brv70185-bib-0133] Planer, R. J. (2023). The evolution of hierarchically structured communication. Frontiers in Psychology 14, 1224324. 37767213 10.3389/fpsyg.2023.1224324 PMC10520573

[brv70185-bib-0134] Planer, R. J. & Kalkman, D. (2021). Arbitrary signals and cognitive complexity. The British Journal for the Philosophy of Science 72, 563–586.

[brv70185-bib-0135] Rapport Munro, E. , Allritz, M. , Schweller, K. , Haun, D. B. M. & Call, J. (2025 *a*). Do chimpanzees (*Pan troglodytes*) attribute preferences to virtual competitors? PLoS One 20, e0329468. 40924714 10.1371/journal.pone.0329468 PMC12419670

[brv70185-bib-0136] Rapport Munro, E. , Koopman, S. E. , Anderson, S. P. , Schweller, K. , Röhr, H. , Kleiman‐Weiner, M. , Lewis, R. , Klein, B. , Allritz, M. , Robinson, L. M. , Dolins, F. L. & Call, J. (2025 *b*). Chimpanzees (*Pan troglodytes*) and bonobos (*Pan paniscus*) chase prey around obstacles in virtual environments. Journal of Comparative Psychology 139(3), 155–177. 40126584 10.1037/com0000402

[brv70185-bib-0137] Rauber, R. , Kranstauber, B. & Manser, M. B. (2020). Call order within vocal sequences of meerkats contains temporary contextual and individual information. BMC Biology 18, 119. 32907574 10.1186/s12915-020-00847-8 PMC7488032

[brv70185-bib-0138] Rendall, D. , Owren, M. J. & Ryan, M. J. (2009). What do animal signals mean? Animal Behaviour 78, 233–240.

[brv70185-bib-0139] Rizzi, L. (2016). Monkey morpho‐syntax and merge‐based systems. Theoretical Linguistics 42, 139–145.

[brv70185-bib-0140] Rutz, C. , Bronstein, M. , Raskin, A. , Vernes, S. C. , Zacarian, K. & Blasi, D. E. (2023). Using machine learning to decode animal communication. Science 381, 152–155. 37440653 10.1126/science.adg7314

[brv70185-bib-0141] Salis, A. , Martin, K. & Girard‐Buttoz, C. (2025). Challenges and new opportunities in deciphering the meaning of corvid call sequences. Animal Cognition 28, 95. 41296061 10.1007/s10071-025-02015-3 PMC12657554

[brv70185-bib-0142] Savage‐Rumbaugh, E. S. , Murphy, J. , Sevcik, R. A. , Brakke, K. E. , Williams, S. L. , Rumbaugh, D. M. & Bates, E. (1993). Language comprehension in ape and child. Monographs of the Society for Research in Child Development 58, 1–252. 8366872

[brv70185-bib-0143] Schamberg, I. , Cheney, D. L. , Clay, Z. , Hohmann, G. & Seyfarth, R. M. (2016). Call combinations, vocal exchanges and interparty movement in wild bonobos. Animal Behaviour 122, 109–116.

[brv70185-bib-0144] Schamberg, I. , Surbeck, M. & Townsend, S. W. (2024). Cross‐population variation in usage of a call combination: evidence of signal usage flexibility in wild bonobos. Animal Cognition 27, 1–7. 39212694 10.1007/s10071-024-01884-4 PMC11364580

[brv70185-bib-0145] Schel, A. M. , Townsend, S. W. , Machanda, Z. , Zuberbühler, K. & Slocombe, K. E. (2013). Chimpanzee alarm call production meets key criteria for intentionality. PLoS One 8, e76674. 24146908 10.1371/journal.pone.0076674 PMC3797826

[brv70185-bib-0146] Schleihauf, H. , Sanford, E. M. , Thompson, B. D. , Zhang, S. , Rukundo, J. , Call, J. , Herrmann, E. & Engelmann, J. M. (2025). Chimpanzees rationally revise their beliefs. Science 390, 521–526. 41166489 10.1126/science.adq5229

[brv70185-bib-0147] Schlenker, P. , Chemla, E. , Schel, A. M. , Fuller, J. , Gautier, J.‐P. , Kuhn, J. , Veselinović, D. , Arnold, K. , Cäsar, C. , Keenan, S. , Lemasson, A. , Ouattara, K. , Ryder, R. & Zuberbühler, K. (2016). Formal monkey linguistics. Theoretical Linguistics 42, 1–90.

[brv70185-bib-0148] Schlenker, P. , Coye, C. , Leroux, M. & Chemla, E. (2023). The ABC‐D of animal linguistics: are syntax and compositionality for real? Biological Reviews 98, 1142–1159. 36960599 10.1111/brv.12944

[brv70185-bib-0149] Schlenker, P. , Salis, A. , Leroux, M. , Coye, C. , Rizzi, L. , Steinert‐Threlkeld, S. & Chemla, E. (2024). Minimal compositionality versus bird implicatures: two theories of ABC‐D sequences in Japanese tits. Biological Reviews 99, 1278–1297. 38545992 10.1111/brv.13068

[brv70185-bib-0150] Scott‐Phillips, T. C. & Blythe, R. A. (2013). Why is combinatorial communication rare in the natural world, and why is language an exception to this trend? Journal of the Royal Society Interface 10, 20130520. 24047871 10.1098/rsif.2013.0520 PMC3785817

[brv70185-bib-0151] Seyfarth, R. M. & Cheney, D. L. (1980). The ontogeny of vervet monkey alarm calling behavior: a preliminary report. Zeitschrift für Tierpsychologie 54, 37–56.

[brv70185-bib-0152] Seyfarth, R. M. & Cheney, D. L. (2010). Production, usage, and comprehension in animal vocalizations. Brain and Language 115, 92–100. 19944456 10.1016/j.bandl.2009.10.003

[brv70185-bib-0153] Seyfarth, R. M. & Cheney, D. L. (2017). The origin of meaning in animal signals. Animal Behaviour 124, 339–346.

[brv70185-bib-0154] Seyfarth, R. M. , Cheney, D. L. & Marler, P. (1980). Monkey responses to three different alarm calls: evidence of predator classification and semantic communication. Science 210, 801–803. 7433999 10.1126/science.7433999

[brv70185-bib-0155] Sievers, C. & Gruber, T. (2016). Reference in human and non‐human primate communication: what does it take to refer? Animal Cognition 19, 759–768. 26971953 10.1007/s10071-016-0974-5

[brv70185-bib-0156] Sigmundson, R. , Girard‐Buttoz, C. , Le Floch, A. , Azaiez, T. S. , McElreath, R. , Zuberbühler, K. , Wittig, R. M. & Crockford, C. (2025). Vocal sequence diversity and length remain stable across ontogeny in a catarrhine monkey (*Cercocebus atys*). Communications Biology 8, 1–13. 40114014 10.1038/s42003-025-07922-2 PMC11926236

[brv70185-bib-0157] Silk, J. B. , Seyfarth, R. M. & Cheney, D. L. (2016). Strategic use of affiliative vocalizations by wild female baboons. PLoS One 11, e0163978. 27783705 10.1371/journal.pone.0163978 PMC5081171

[brv70185-bib-0158] Skyrms, B. (2010). Signals: Evolution, Learning, and Information. Oxford University Press, Oxford, UK.

[brv70185-bib-0159] Stewart, I. , McElwee, J. & Ming, S. (2013). Language generativity, response generalization, and derived relational responding. The Analysis of Verbal Behavior 29, 137–155. 23814374 10.1007/BF03393131 PMC3659503

[brv70185-bib-0160] Suzuki, T. N. & Matsumoto, Y. K. (2022). Experimental evidence for core‐merge in the vocal communication system of a wild passerine. Nature Communications 13, 5605. 10.1038/s41467-022-33360-3 PMC9509327 36153329

[brv70185-bib-0162] Suzuki, T. N. , Wheatcroft, D. & Griesser, M. (2016). Experimental evidence for compositional syntax in bird calls. Nature Communications 7, 10986. 10.1038/ncomms10986 PMC4786783 26954097

[brv70185-bib-0161] Suzuki, T. N. , Wheatcroft, D. & Griesser, M. (2018). Call combinations in birds and the evolution of compositional syntax. PLoS Biology 16, e2006532. 30110321 10.1371/journal.pbio.2006532 PMC6093598

[brv70185-bib-0163] Suzuki, T. N. & Zuberbühler, K. (2019). Animal syntax. Current Biology 29, R669–R671. 31336078 10.1016/j.cub.2019.05.045

[brv70185-bib-0164] Tanner, J. E. & Perlman, M. (2017). Moving beyond ‘meaning’: gorillas combine gestures into sequences for creative display. Language & Communication 54, 56–72.

[brv70185-bib-0165] Taylor, D. , Clay, Z. , Dahl, C. D. , Zuberbühler, K. , Davila‐Ross, M. & Dezecache, G. (2022). Vocal functional flexibility: what it is and why it matters. Animal Behaviour 186, 93–100.

[brv70185-bib-0166] Templeton, C. N. , Greene, E. & Davis, K. (2005). Allometry of alarm calls: black‐capped chickadees encode information about predator size. Science 308, 1934–1937. 15976305 10.1126/science.1108841

[brv70185-bib-0167] Townrow, L. A. & Krupenye, C. (2025). Bonobos point more for ignorant than knowledgeable social partners. Proceedings of the National Academy of Sciences 122, e2412450122. 10.1073/pnas.2412450122 PMC11831142 39899718

[brv70185-bib-0168] Townsend, S. W. , Engesser, S. , Stoll, S. , Zuberbühler, K. & Bickel, B. (2018). Compositionality in animals and humans. PLoS Biology 16, 1–7. 10.1371/journal.pbio.2006425 PMC6093600 30110319

[brv70185-bib-0169] Truswell, R. (2017). Dendrophobia in bonobo comprehension of spoken English. Mind & Language 32, 395–415.

[brv70185-bib-0170] Valet, A. , Bacquelé, Q. , Girard‐Buttoz, C. , Wittig, R. M. & Crockford, C. (2025). AI decodes chimpanzee vocabulary items. In The Thirty‐Ninth Annual Conference Proceedings on Neural Information Processing Systems (NeurIPS). (eds D. Belgrave , C. Zhang , H. Lin , R. Pascanu , P. Koniusz , M. Ghassemi , N. Chen ).

[brv70185-bib-0171] Vernes, S. C. , Janik, V. M. , Fitch, W. T. & Slater, P. J. B. (2021). Vocal learning in animals and humans. Philosophical Transactions of the Royal Society, B: Biological Sciences 376, 20200234. 10.1098/rstb.2020.0234 PMC8422595 34482718

[brv70185-bib-0172] Walsh, S. L. , Townsend, S. W. , Morgan, K. & Ridley, A. R. (2019). Investigating the potential for call combinations in a lifelong vocal learner. Ethology 125(6), 362–368.

[brv70185-bib-0173] Watson, S. K. , Filippi, P. , Gasparri, L. , Falk, N. , Tamer, N. , Widmer, P. , Manser, M. & Glock, H.‐J. (2022). Optionality in animal communication: a novel framework for examining the evolution of arbitrariness. Biological Reviews 97, 2057–2075. 35818133 10.1111/brv.12882 PMC9795909

[brv70185-bib-0174] Wegdell, F. , Hammerschmidt, K. & Fischer, J. (2019). Conserved alarm calls but rapid auditory learning in monkey responses to novel flying objects. Nature Ecology & Evolution 3, 1039–1042. 31133723 10.1038/s41559-019-0903-5

[brv70185-bib-0175] West, S. A. , Fisher, R. M. , Gardner, A. & Kiers, E. T. (2015). Major evolutionary transitions in individuality. Proceedings of the National Academy of Sciences 112, 10112–10119. 10.1073/pnas.1421402112 PMC4547252 25964342

[brv70185-bib-0176] Wilson, B. , Spierings, M. , Ravignani, A. , Mueller, J. L. , Mintz, T. H. , Wijnen, F. , van der Kant, A. , Smith, K. & Rey, A. (2020). Non‐adjacent dependency learning in humans and other animals. Topics in Cognitive Science 12, 843–858. 32729673 10.1111/tops.12381 PMC7496455

[brv70185-bib-0177] Xie, J. , Zhong, Y. , Zhang, J. , Liu, S. , Ding, C. & Triantafyllopoulos, A. (2023). A review of automatic recognition technology for bird vocalizations in the deep learning era. Ecological Informatics 73, 101927.

[brv70185-bib-0178] Zaccarella, E. , Schell, M. & Friederici, A. D. (2017). Reviewing the functional basis of the syntactic merge mechanism for language: a coordinate‐based activation likelihood estimation meta‐analysis. Neuroscience & Biobehavioral Reviews 80, 646–656. 28743620 10.1016/j.neubiorev.2017.06.011

[brv70185-bib-0179] Zuberbühler, K. (2019). Evolutionary roads to syntax. Animal Behaviour 151, 259–265.

[brv70185-bib-0180] Zuberbühler, K. (2020). Syntax and compositionality in animal communication. Philosophical Transactions of the Royal Society, B: Biological Sciences 375, 20190062. 10.1098/rstb.2019.0062 PMC6895557 31735152

[brv70185-bib-0181] Zuberbühler, K. & Bickel, B. (2022). Transition to language: from agent perception to event representation. WIREs Cognitive Science 13, e1594. 35639563 10.1002/wcs.1594 PMC9786335

[brv70185-bib-0182] Zuberbühler, K. , Cheney, D. L. & Seyfarth, R. M. (1999). Conceptual semantics in a nonhuman primate. Journal of Comparative Psychology 113, 33–42.

